# USP35 mitigates endoplasmic reticulum stress‐induced apoptosis by stabilizing RRBP1 in non‐small cell lung cancer

**DOI:** 10.1002/1878-0261.13112

**Published:** 2021-10-18

**Authors:** Wenqing Wang, Meixia Wang, Yi Xiao, Yige Wang, Lijuan Ma, Lulu Guo, Xinyue Wu, Xiaoyan Lin, Pengju Zhang

**Affiliations:** ^1^ Department of Biochemistry and Molecular Biology Shandong University School of Basic Medical Sciences Jinan China; ^2^ Department of Internal Medicine Qingdao Fuwai Cardiovascular Hospital China; ^3^ Eppley Institute for Research in Cancer and Allied Diseases Fred & Pamela Buffet Cancer Center University of Nebraska Medical Center Omaha NE USA; ^4^ Department of Pathology Shandong Provincial Hospital Affiliated to Shandong University Jinan China

**Keywords:** deubiquitinating enzymes, endoplasmic reticulum stress, non‐small cell lung cancer, ribosome binding protein 1, ubiquitin‐specific‐processing protease 35

## Abstract

Deubiquitinating enzymes (DUBs) serve to maintain cellular homeostasis via protein ubiquitination and exert diverse regulatory functions in cancers and other diseases. Much progress has been made in characterizing biological roles of DUBs over the decades, yet the specific functions of many subclass members remain largely unexplored. It was not until recent years that the role of ubiquitin‐specific‐processing protease 35 (USP35) in cancers began to be understood. Here, we focus on delineating the roles and underlying mechanisms of USP35 in non‐small cell lung cancer (NSCLC). The isobaric tags for relative and absolute quantitation (iTRAQ) comparative proteomic approach were employed to identify differentially expressed proteins (DEPs) in H1299 cells induced by *USP35* overexpression or silencing. Among the potential interactome of USP35, ribosome‐binding protein 1 (RRBP1), a membrane‐bound protein in endoplasmic reticulum (ER), captured our attentions. RRBP1 expression was found to positively correlate with USP35 levels in both genetically modified cells and human NSCLC tissues. Concordantly, both *RRBP1* expression and *USP35* expression were found to positively correlate with poor prognoses in lung adenocarcinoma patients. At the molecular level, USP35 was verified to directly interact with RRBP1 to prevent it from proteasomal‐dependent degradation. Functionally, USP35 alleviated ER stress‐induced cell apoptosis by stabilizing RRBP1 in NSCLC cells. Collectively, these findings indicate that USP35 plays a critical role in resisting ER stress‐induced cell death through deubiquitinating RRBP1, hence providing a rationale to target the USP35‐RRBP1 axis as an alternative therapeutic option for NSCLC.

## Graphical abstract

Ribosome‐binding protein 1 (RRBP1) was found to positively correlate with ubiquitin‐specific protease 35 (USP35) levels by proteomic analysis, which was validated in genetically modified cells and human non‐small cell lung cancer (NSCLC) tissues. USP35 was verified to interact with RRBP1 and prevent it from proteasomal‐dependent degradation. USP35 alleviated endoplasmic reticulum stress‐induced cell apoptosis by stabilizing RRBP1 in NSCLC cells.

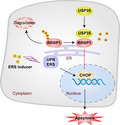

**Abbreviations**
DEPs: differentially expressed proteins
DUBs: deubiquitinating enzymes
ER: endoplasmic reticulum
iTRAQ: the isobaric tags for relative and absolute quantitation
NSCLC: non‐small cell lung cancer
RRBP1: ribosome‐binding protein 1
shRNAs: short hairpin RNAs
TM: tunicamycin
TUNEL: terminal dUTP nick‐end labeling
UPR: unfolded protein response
USP35: ubiquitin‐specific‐processing protease 35

## 1 Introduction

Lung cancer is the leading cause of cancer death worldwide with incidence rates steadily remaining the top three over the decades in both men and women [1]. Accounting for 85% of all lung cancer at diagnosis, non‐small cell lung cancer (NSCLC) is the most common type of lung cancer [2]. Although 5‐year survival rate is over 50% for localized lung cancer, most cancers have already spread at first diagnosis with overall survival rate of < 20% and the situation gets far worse for the cases diagnosed with distant metastases [3]. For years, much effort has been made to expand treatment options for a variety of cancers, and indeed, advancements in immunotherapies and targeted therapies have created therapeutic landscape for the NSCLC patients of all stages [4, 5, 6, 7]. However, only part of the patients are proper candidates for current molecular therapies and complex and heterogeneous nature of cancer pushes us to explore more of the molecular mechanisms of oncogenesis in order to better the therapeutic options for a wider range of cancer population. Therefore, it is imperative that we have a more comprehensive understanding of the molecular mechanisms of NSCLC to pave the way for discovering powerful anticancer arsenals in combating NSCLC.

Ubiquitination is one of the most important post‐translational modifications that influence protein stability, subcellular localization, molecular interaction, and activity [8, 9, 10]. Ubiquitin is a highly conserved and widely distributed small molecule protein consisting of 76 amino acid residues. The process of protein ubiquitination refers to covalent addition of ubiquitin(s) to the lysine residue of target protein, which is mediated by three enzymes: ubiquitin‐activating enzyme (E1), ubiquitin‐conjugating enzyme (E2), and ubiquitin ligase (E3) [11, 12, 13]. Protein ubiquitination could be reversed by deubiquitination that removes ubiquitins from the substrate catalyzed by deubiquitinating enzymes (DUBs) [14]. A dynamic balance between ubiquitination and deubiquitination is crucial for normal cellular functions.

The human genome encodes approximately 100 DUBs which participate in almost all life activities such as cell cycle, proliferation, apoptosis, signal transduction, and DNA damage repair [15, 16, 17]. According to the feature of the catalytic domain, DUBs can be subgrouped into seven distinct families: ubiquitin‐specific proteases (USPs), ubiquitin C‐terminal hydrolases (UCHs), ovarian tumor proteases (OTUs), Machado‐Joseph disease proteases (MJDs), Jab1/Mov34/Mpr1 (JAMM) metalloproteases, MIU‐containing novel DUB (MINDY), and zinc finger‐containing ubiquitin peptidase 1 (ZUP1) [18]. To date, accumulating evidence has suggested that altered DUB activity and expression are closely related to the occurrence and development of several cancers, thus making DUBs promising candidates for anticancer drug development [19, 20, 21]. Despite great progress in identifying and understanding the roles of DUBs during the past decades, the characteristics of many DUBs stay largely unknown.

Ubiquitin‐specific‐processing proteases 35 (USP35) belong to the peptidase C19 family and its roles are poorly characterized. Recently, emerging evidence suggests that USP35 is involved in cell cycle regulation, cell fate, and cancer development. USP35 regulates cell mitosis by deubiquitinating Aurora B and prevents its degradation from APC/CDH1 [22]. USP35 is reported to regulate MFN2 abundance and PARK2‐mediated mitophagy [23]. Our previous work has found that USP35 impedes tumor growth through inhibiting NF‐κB activation by stabilizing ABIN‐2 [24]. Besides, USP35 has been shown to be upregulated in ovarian cancer and contribute to chemoresistance via activating STING‐TBK1‐IRF3 pathway [25]. Given the underlying roles of USP35 in cancers and the gap of current knowledge of this molecule, we aim to understand the biological functions of USP35, specifically in NSCLC.

Ribosome‐binding protein 1(RRBP1) is an endoplasmic reticulum (ER) membrane protein and plays important roles in ribosome binding, nascent protein translocation as well as in ER stress and unfolded protein response (UPR) [26]. So far, a number of studies have shown that RRBP1 is highly expressed in multiple malignancies including cervical squamous cell carcinoma, esophageal carcinoma, ovarian cancer, prostate cancer, and colorectal cancer [27, 28, 29, 30, 31]. Moreover, it is reported that elevated RRBP1 closely correlates with cancer progression and poor prognosis. For example, *RRBP1* mRNA and protein expressions are significantly increased in invasive breast cancer compared with normal tissues [32]. Specifically, RRBP1 is a potential prognostic indicator for Her‐2‐positive breast cancer patients [33]. Bladder cancer patients with high RRBP1 expression display shorter overall survival. Meanwhile, RRBP1 overexpression promotes migration and invasion of bladder cancer cells [34]. Also, RRBP1 is reported to be overexpressed in lung cancer and its overexpression alleviates ER stress via enhancing GRP78 expression in lung cancer cells [26]. All such evidence supports that deregulation of RRBP1 contributes to cancer development and progression.

In the present study, we first used the isobaric tags for relative and absolute quantitation (iTRAQ) comparative proteomic approach to globally screen interactome of USP35 in NSCLC cells and identified RRBP1 as a substrate of interest. We confirmed that USP35 directly bound to and deubiquitinated RRBP1, thus preventing it from proteasome‐dependent degradation and resulted in RRBP1 enrichment in the cells. Functionally, we found that USP35 overexpression attenuated ER stress‐induced cell apoptosis by stabilizing RRBP1 in NSCLC cells. Finally, positive correlation between USP35 and RRBP1 in human NSCLC tissues, as well as positive correlation between USP35 or RRBP1 and shorter overall survival of lung adenocarcinoma patients was confirmed. Overall, we disclosed a novel role of USP35 in regulating ER stress by stabilizing RRBP1, thus presenting USP35‐RRBP1 axis as a novel target for NSCLC treatment.

## 2 Materials and methods

### 2.1 Cell culture and cell transfection

The human NSCLC cell lines A549, PC9, and H1299 were purchased from the Cell Bank of the Chinese Academy of Science (Shanghai, China) and cultured in RPMI‐1640 medium with 10% FBS. Human embryonic kidney 293T (HEK293T) cells were purchased from the American Type Culture Collection (Manassas, VA, USA) and maintained in Dulbecco's modified Eagle's medium containing 10% FBS. All cells were cultured at 37 °C with 5% CO_2_ atmosphere and were resuscitated every 3 months and tested negative for mycoplasma contamination.

For all transfection procedures, standard protocols were followed in accordance with manufacturer's instructions using Lipofectamine 2000 (Invitrogen, Waltham, MA, USA).

### 2.2 Antibodies and reagents

The antibodies used in our experiments are listed in Table S1. Tunicamycin (TM, ER stress inducer) was purchased from Solarbio (Beijing, China) and stocked at concentration of 2 mm in dimethyl‐sulfoxide (DMSO). MG132 (proteasome inhibitor), and cycloheximide (CHX, protein synthesis inhibitor) was purchased from Calbiochem (Darmstadt, Germany) and stocked at concentration of 20 mm and 50 mg·mL^−1^ in DMSO, respectively. Radezolid (RRBP1 inhibitor) was purchased from APExBIO (Houston, TX, USA) and stocked at the concentration of 10 mm in DMSO. 0.5, 1, and 2 µm TM were used for 48 h or 2 µm TM was used for 24, 48, and 72 h to induce ER stress. Ten micromolar MG132 was used for 6 h prior to harvesting the cells, and 50 μg·mL^−1^ CHX was used for indicated time to detect protein degradation. Ten micromolar Radezolid was used to inhibit RRBP1 expression.

### 2.3 Expression plasmids

Human wild‐type (WT) *USP35* expression vectors pCMV‐3XHA‐USP35 WT, pCMV‐3XMyc‐USP35 WT, and pCMV‐3XFlag‐USP35 WT were previously constructed by our laboratory [24]. The catalytically inactive mutant of USP35, pCMV‐3XHA‐USP35 C450A, was generated by site‐directed mutagenesis using the Stratagene QuikChange Site‐Directed Mutagenesis Kit (Agilent, Shanghai, China). *USP35* cDNA was subcloned into pLVX‐IRES‐Puro vector for lentivirus production. The specific short hairpin RNAs (shRNAs) targeting human *USP35* and noneffective scrambled shRNA (GTTCTCCGAACGTGTCACGT) were synthesized and cloned into pLKO.1‐TRC vector (GenePharma, Shanghai, China). SiRNAs specifically targeting *USP35* were synthesized by GeneChem Co (Shanghai, China). Specific gRNAs for *USP35* were synthesized and cloned into H6825 pLenti‐U6‐spgRNA v2.0‐CMV‐sfGFP‐P2A‐3Flag‐spCas9 vector (Obio Technology, Shanghai, China). PcDNA3.1‐Flag‐RRBP1 plasmid was purchased from Fenghbio (Hunan, China). SiRNAs specifically targeting RRBP1 were purchased from GenePharma.

### 2.4 siRNA/shRNA target sequences

USP35‐shRNA‐3: 5′‐GCTGAGTTGGGCTCTTC TAGA‐3′
USP35‐shRNA‐4: 5′‐GCGTCTGACTTCAGACATTG‐3′
USP35‐siRNA‐1: 5′‐GGGAAGATCTGATGATGTT‐3′
USP35‐ siRNA‐2: 5′‐CCAAGAGGAAGGATGGTAC‐3′
RRBP1‐siRNA‐1: 5′‐GTGAAGCATCTCGAAGAGATT‐3′
RRBP1‐siRNA‐2: 5′‐CCTAATGGGAAGATACCTGAT‐3′
RRBP1‐siRNA‐3: 5′‐GCATGTCGGTTACAAGAAGAA‐3′
USP35‐gRNA‐1: 5′‐ACTCGGCGAAGACGTCGGGGTGG‐3′
USP35‐gRNA‐2: 5′‐CAGCAGCGCGAACACCTCGTCGG‐3′
USP35‐gRNA‐3: 5′‐CACCCGCGCTGTGTGCCCGACGG‐3′

### 2.5 Establishment of USP35 stable overexpression and knockdown cell lines

To generate *USP35* stable overexpressing and knockdown cell lines, the aforementioned lentiviral constructs expressing *USP35* cDNA or *USP35* shRNAs as well as their corresponding control constructs were cotransfected into HEK293T cells with lentivirus packaging plasmids psPAX2 and pMD2 (Genechem Co). After 48 h infection, the viral supernatants were collected and filtered with 0.45 µm syringe filters. Then, the indicated NSCLC cells were cultured with the viral supernatants containing 4 µg·mL^−1^ polybrene (Millipore/Chemicon, Billerica, MA, USA) for 48 h, followed by continuous culture in complete medium containing 2 µg·mL^−1^ puromycin for another 48 h. The expression of USP35 was further confirmed by western blot.

### 2.6 iTRAQ labeling and LC‑MS/MS analysis

**Fig. 1:**
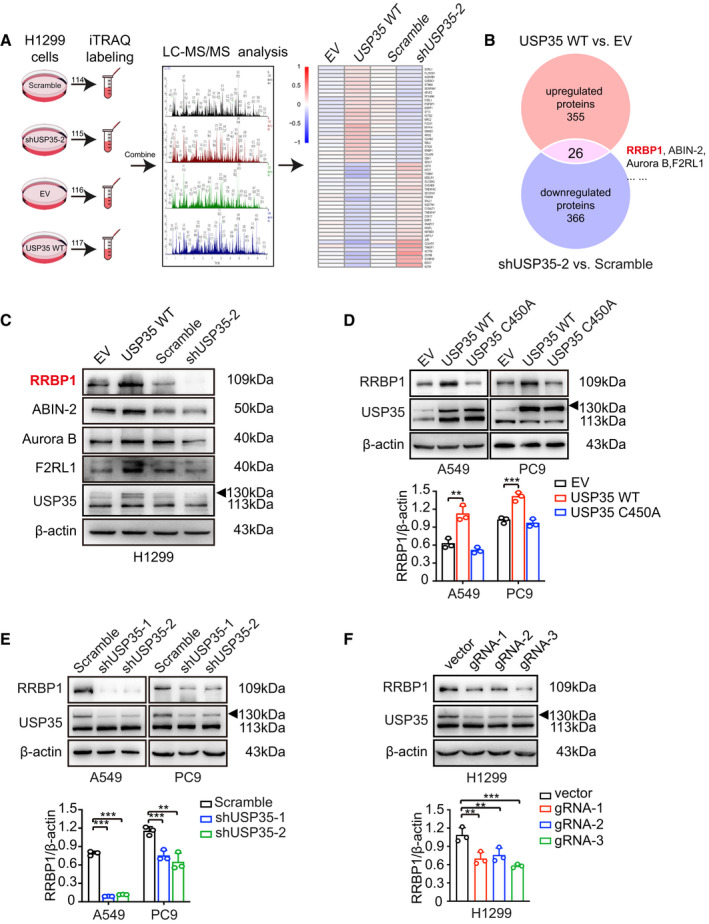
USP35 upregulates RRBP1 protein level. (A) Flowchart of proteomic analysis was illustrated. (B) Twenty‐six overlapped proteins were identified to be increased in wild‐type USP35 overexpression group (USP35 WT) and decreased in shUSP35‐2 group through Venn diagrams software (available online: http://bioinformatics.psb.ugent.be/webtools/Venn/). (C) The indicated DEPs were confirmed by western blot. (D–F) Expression of RRBP1 was detected by western blot in USP35 wild‐type or catalytically inactive mutant overexpression (WT and C450A) A549 and PC9 cells (D), shRNAs mediated USP35 knockdown A549 and PC9 cells (E) and gRNA mediated USP35 knockdown H1299 cells (F). All data are presented by mean ± SD. **P < 0.01, ***P < 0.001 based on the Student *t*‐test. All results are representatives of three independent experiments.

Proteomic analysis was performed to identify proteins that were differentially expressed in *USP35* overexpressed or silenced H1299 cells. The procedure of the experiment was performed in a manner similar to that in previous studies [35]. In brief, protein samples in each group were extracted using SDT lysis buffer and digested by the means of filter‐aided sample preparation [36]. ITRAQ tags 113, 114, 115, and 116 (Applied Biosystems, Foster City, CA, USA) were used to label each sample containing 100 µg peptides. Then, the labeled peptides from each group were mixed at equal ratios and further fractionated using Agilent 1260 infinity II high‐performance liquid chromatography. The fractions were separated using a Thermo Easy‐nLC binary buffer system and then subjected to a Q‐Exactive mass spectrometer for protein identification. Figure 1A shows the flow chart of proteomic analysis.

mascot 2.5 (Matrix Science, Boston, MA, USA) and proteome discoverer 2.1 software (Thermo Fisher Scientific, Waltham, MA, USA) were used for the identification and quantitative analysis of proteins based on data from the Database (uniprot_HomoSapiens_20161205.fasta). Proteins with differential ratio of more than 1.2 (up/down) and *P*‐value < 0.05 were considered as differentially expressed proteins (DEPs).

### 2.7 Western blot

Total cell extracts were prepared in RIPA buffer (Thermo Fisher Scientific, Waltham, MA, USA) supplemented with protease inhibitors (Roche, Indianapolis, IN, USA) and phosphatase inhibitors (Solarbio). Protein samples were separated by 8–12% SDS/PAGE and electrically transferred to polyvinylidene fluoride membrane (Millipore, Billerica, MA, USA). Then, the membrane was immunoblotted with the indicated antibodies.

### 2.8 Co‐immunoprecipitation

For co‐immunoprecipitation (Co‐IP), total cell extracts were prepared in IP lysis buffer (Vazyme, Nanjing, China) supplemented with protease inhibitor (Roche, Indianapolis, IN, USA). Aimed proteins were immunoprecipitated with the indicated target‐specific antibody (1 µg) overnight at 4 °C. Next day, the samples were incubated with 40 µL PureProteome™ Protein A/G Mix Magnetic Beads (Millipore) for 30 min at room temperature and then were washed with PBS containing 0.1% Tween 20 (pH 7.4) for five times before denaturation. Finally, the immunoprecipitated samples were identified by western blot with their corresponding primary antibodies.

### 2.9 Quantitative real‐time PCR

Total RNAs were isolated using the TRIzol reagent (Invitrogen, Carlsbad, CA, USA). Reverse transcription was carried out using the SuperScript III Reverse Transcriptase kit (Invitrogen, Carlsbad, CA, USA). Real‐time PCR was performed using qPCR SYBR Green master mix (Takara Bio, Shiga, Japan) on a CFX Connect Real‐time PCR Detection System (Bio‐Rad, Hercules, CA, USA). β‐actin was used as an internal control for RNA expression normalization using the $2^{‐\Delta \Delta C_{t}}$ method. The sequences of the primer pairs were:

*β‐actin* forward: 5′‐AGTTGCGTTACACCCTTTCTTG‐3′
*β‐actin* reverse: 5′‐CACCTTCACCGTTCCAGTTTT‐3′
*RRBP1* forward: 5′‐AGTTCGGACCAGGTGAGGGAGCAC‐3′
*RRBP1* reverse: 5′‐GCGTCTTCAGCTGAACGGGGTC‐3′

### 2.10 Immunofluorescence assay

The cells were seeded on glass coverslips in 24‐well plates. At 70% confluency, they were fixed in 4% paraformaldehyde for 15 min at 4 °C, followed by permeabilization with 0.2% Triton X‐100 in PBS (Solarbio). After blockage with 5% skimmed milk in PBS for 1 h at room temperature, cells were incubated with indicated primary antibodies at 1 : 50 dilution overnight at 4 °C in PBST (Solarbio). Then, cells were washed three times with PBST and incubated with rhodamine (TRITC)‐conjugated or fluorescein isothiocyanate (FITC)‐conjugated secondary antibodies for 2 h at 37 °C. After washing them with PBST for three times, slips were mounted with antifade mounting medium containing 2 µg·mL^−1^ of 4,6‐diamidine‐2‐phenylindole dihydrochloride (DAPI; Beyotime, Haimen, China). Fluorescent images were collected on a confocal microscope (Andor, Belfast, UK). All images were taken with the same exposure time and processed with same standard.

### 2.11 Flow cytometry analysis

Cell apoptosis was detected using Annexin V‐FITC/PI apoptosis detection kit (Vazyme), and fluorescence‐activated cell sorter (FACS) was used for quantification. Briefly, 2 × 10^5^ cells were seeded in 6‐well plates and treated with 2 µm TM or DMSO for 48 h. Then, cells were collected and resuspended in 100 μL 1× binding buffer, followed by staining with 5 μL Annexin V‐FITC and 5 μL propidium idodide (PI) for 10 min at room temperature in the dark. The stained samples were analyzed by CytoFLex S (Beckman Coulter, Brea, CA, USA) using cytexpert Software (Beckman Coulter) within an hour. Theoretically, the cells could be divided into the following four groups according to the fluorescence staining through flow cytometry: nonapoptotic cells (Annexin V‐FITC‐negative/PI‐negative), early apoptotic cells (Annexin V‐FITC‐positive/PI‐negative), late apoptotic/necrotic cells (Annexin V‐FITC‐positive/PI‐positive), and dead cells (Annexin V‐FITC‐negative/PI‐positive).

### 2.12 Terminal dUTP nick‐end labeling assay

To detect DNA fragmentation, terminal dUTP nick‐end labeling (TUNEL) staining was performed using TUNEL BrightGreen Apoptosis Detection Kit (Vazyme) according to manufacturer's instructions. In short, well‐fixed and permeabilized cells on the slide were labeled in 50 μL of TdT reaction buffer at 37 °C for 1 h in a moist, dark chamber. Counterstaining was performed with antifade mounting medium containing 2 µg·mL^−1^ DAPI (Beyotime). Then, the samples were analyzed under a fluorescence microscope (Axio Scope A1, Wuxi, China) with standard light filters and zen connected software (Oberkochen, Germany).

### 2.13 Immunohistochemistry and scoring

Forty‐five NSCLC tissues were obtained from surgical patients in Shandong provincial hospital (Jinan, China) with the understanding and written consent of patients. The study methodologies were approved by the Shandong University Ethical Review Board and conformed to the standards set by the Declaration of Helsinki. The IHC analysis of USP35 and RRBP1 expression in clinical samples was performed as previously described [37]. The sections were incubated with USP35 (1 : 100; Abcam, Cambridge, UK) and RRBP1 (1 : 100; Proteintech, Chicago, IL, USA) antibodies. The scoring methods were described in our previous study [38]. The scoring results were analyzed by two experienced pathologists.

### 2.14 Statistical analysis

graphpad prism 8 software (La Jolla, CA, USA) was used for statistical analysis. All data were shown as mean ± SD from at least three independent experiments. A two‐tailed, unpaired Student's *t*‐test was used for comparisons between two groups. The one‐way ANOVA test was used to assess possible correlations between USP35/RRBP1 expression and clinicopathologic factors. *P* < 0.05 was considered statistically significant.

## 3 Results

### 3.1 Global proteomic screening identifies RRBP1 as a novel target of USP35

To investigate the potential deubiquitination substrates of USP35, isobaric tags for relative and absolute quantitation (iTRAQ), combined with liquid chromatography/tandem mass spectrometric (LC‐MS/MS) analysis, were used to identify proteins that were differentially expressed in H299 cells with ectopic *USP35* overexpression (H1299‐USP35) or knockdown compared with their specific parental cells. The strategy of iTRAQ analysis between the two paired groups of cells (H1299‐USP35 *vs* H1299‐EV; H1299‐shUSP35 *vs* H1299‐shNC) was presented in Fig. 1A. Fold changes > 1.2 were set to distinguish up‐ or downregulated differential expression proteins (DEPs) with *P* values < 0.05. Using mascot 2.5 (Matrix Science, Boston, MA, USA) and proteome discoverer 2.1 (Thermo Fisher Scientific, Waltham, MA, USA), a total of 7543 proteins and 82 826 peptides were identified (global false discovery rate < 0.01). Among them, 355 proteins were upregulated and 323 proteins were downregulated in H1299 cells transfected with *USP35* expression vector (H1299‐USP35) compared with the control cells transfected with empty vector (H1299‐EV) (Table S2). Meanwhile, 208 upregulated and 366 downregulated proteins were identified in H1299 cells transfected with *USP35*‐specific targeting shRNAs (H1299‐shUSP35‐2) compared with the control cells transfected with nonspecific scrambled RNAs (H1299‐scramble) (Table S3). To further clarify the possible targets regulated by USP35, the overlapping proteins between USP35‐upregulated proteins and shUSP35‐downregulated proteins were screened and a total of 26 overlapping proteins were identified, of which RRBP1 displayed a significant altered expression (1.63‐fold change, *P* = 0.00027; 0.79‐fold change, *P* = 0.01) (Fig. 1B, Table S4). Then, the differential expression of RRBP1 and other identified protein, including ABIN‐2 and Aurora B, were validated by western blot analysis (Fig. 1C, Fig. S1A). To further validate the regulation of USP35 on RRBP1 in NSCLC cells, wild‐type USP35 (WT), USP35 catalytic dead mutant C450A, and empty vector plasmids were transfected into A549 and PC9 cells, respectively. Ectopic expression of wild‐type USP35 strongly increased the expression of RRBP1, while ectopic expression of USP35 C450A mutant had little effect on the expression of RRBP1 (Fig. 1D). Next, whether inhibition of USP35 could affect RRBP1 expression was determined. Specific shRNAs targeting *USP35* and scrambled shRNA were introduced into A549 and PC9 cells by lentiviral transduction. USP35 silencing significantly suppressed the expression of RRBP1 (Fig. 1E). Consistently, decreased RRBP1 protein levels were also observed in *USP35* knockout cell lines by independent *USP35* gRNAs introduction (Fig. 1F). As expected, no change in *RRBP1* mRNA level was observed after USP35 silencing or overexpressing in aforementioned cell lines (Fig. S1B,C). Together, these results proved that RRBP1 expression could be upregulated by USP35 at post‐transcriptional level.

### 3.2 USP35 positively regulates RRBP1 stability in a proteasome‐dependent manner

**Fig. 2:**
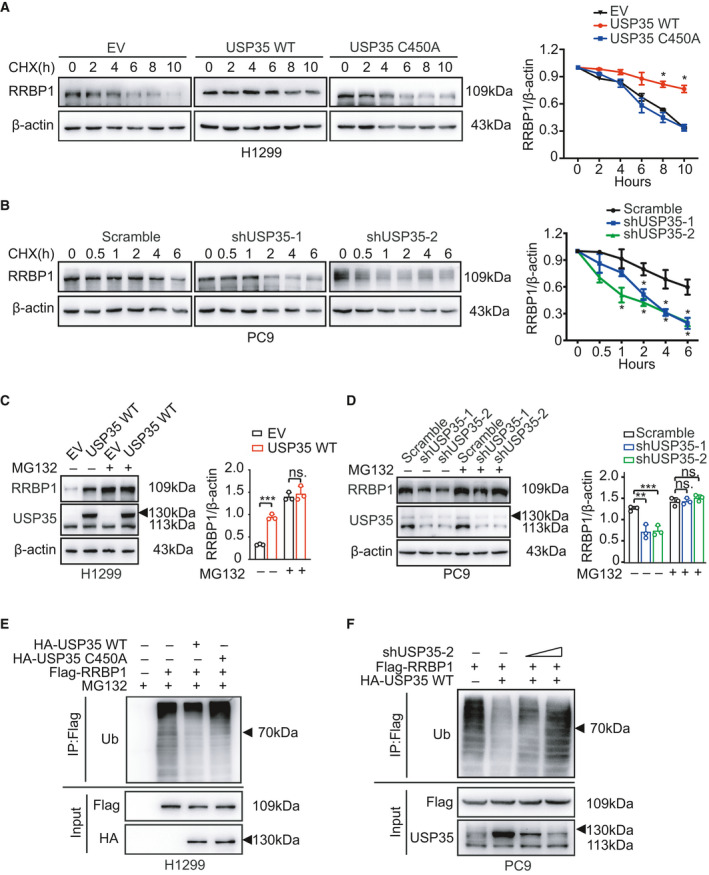
USP35 deubiquitinates and stabilizes RRBP1. (A, B) H1299 cells stably expressing wild‐type USP35 (USP35 WT) and catalytically inactive form, USP35 C450A as well as empty vector (EV) (A), and PC9 cells with stably expressing USP35‐specific shRNAs (shUSP35‐1, shUSP35‐2) and scramble RNA (B) were treated with 50 μg·mL^−1^ cycloheximide (CHX) for 0, 2, 4, 6, 8, 10 h (A) or for 0, 0.5, 1, 2, 4, and 6 h (B). Expressions of RRBP1 were detected by western blot. Quantitative analyses of CHX chase data were shown in the graphs. (C, D) USP35 overexpressed (USP35 WT) H1299 cells and the control group (EV) (C), and USP35 knockdown (shUSP35‐1 and shUSP35‐2) PC9 cells and the control group (Scramble) (D) were treated with or without 10 µm MG132 for 6 h. Expressions of RRBP1 were detected by western blot. Quantitative analyses were shown in the graphs. (E) H1299 cells were transfected or cotransfected with Flag‐RRBP1 alone or along with HA‐USP35 WT or HA‐USP35 C450A. Cell lysates were immunoprecipitated with anti‐Flag antibody, followed by immunoblotting with anti‐Ub antibody. (F) PC9 cells were transfected or cotransfected with Flag‐RRBP1 alone or along with HA‐USP35 WT or in combination with HA‐USP35 WT and 2 or 4 µg shUSP35‐2. Cell lysates were immunoprecipitated with anti‐Flag antibody, followed by immunoblotting with anti‐Ub antibody. All data are presented by mean ± SD. *P < 0.05, **P < 0.01, ***P < 0.001 based on the Student *t*‐test. All results are representatives of three independent experiments.

Considering the functions of DUBs in stabilizing their target proteins, we first investigated the effect of USP35 on protein stability of RRBP1. Cycloheximide (CHX) chase assay was performed to measure RRBP1 degradation in different USP35 expression circumstances. As shown in Fig. 2A, overexpression of wild‐type USP35 (WT), but not the catalytic dead mutant (C450A), decreased degradation of RRBP1 in H1299 cells. Meanwhile, USP35 knockdown led to a more rapid degradation of RRBP1 protein than that observed in control cells (Fig. 2B). These results indicated that USP35 increased RRBP1 expression through preventing its degradation. Next, H1299 cells with ectopic USP35 overexpression and PC9 cells with USP35 silencing were treated with proteasome inhibitor MG132 to evaluate whether USP35 could protect RRBP1 from proteasome‐dependent degradation. Notably, RRBP1 protein levels were increased in all the indicated cells and USP35 overexpression failed to accumulate more RRBP1 than its control in the presence of MG132 (Fig. 2C). Consistently, USP35 knockdown‐induced decrease of RRBP1 was also rescued by MG132 (Fig. 2D). These data suggested that USP35 stabilized RRBP1 by inhibiting its proteasomal degradation.

Since DUBs usually stabilize their substrates through promoting their deubiquitination, we then investigated whether USP35 could influence the ubiquitination of RRBP1. Indeed, overexpression of wild‐type USP35, but not the C450A mutant, decreased poly‐ubiquitination of both exogenous RRBP1 and endogenous RRBP1 in H1299 cells (Fig. 2E, Fig. S2A). Consistently, USP35 silencing in PC9 cells led to a significant increase of poly‐ubiquitination of exogenous RRBP1 (Fig. 2F, Fig. S2B). Taken together, these results indicated that USP35 promoted deubiquitination of RRBP1, thus preventing it from proteasomal degradation.

### 3.3 USP35 physically interacts with RRBP1

**Fig. 3:**
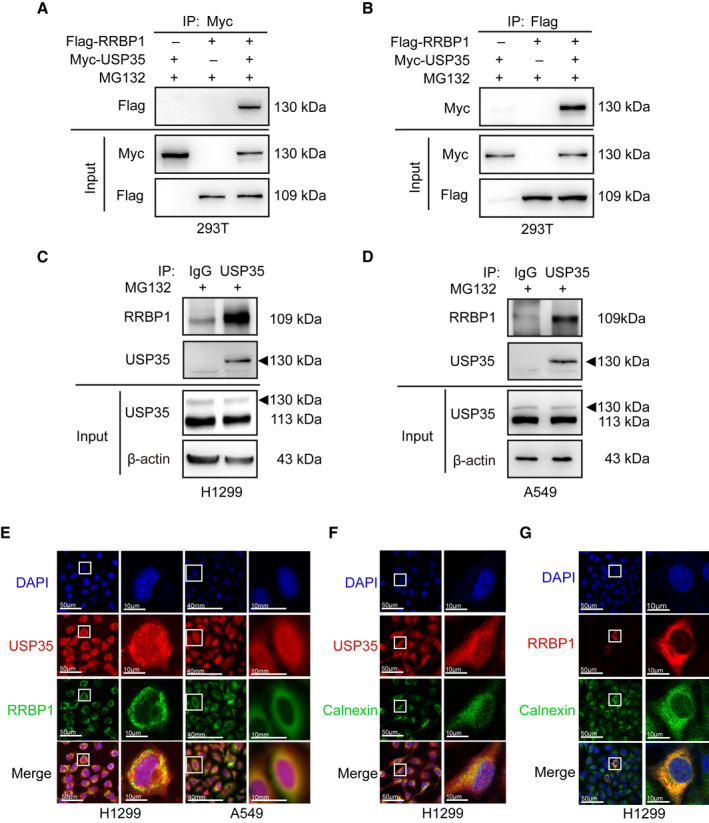
USP35 interacts with RRBP1. (A, B) HEK293T cells were cotransfected with Flag‐RRBP1 and Myc‐USP35. Cell lysates were immunoprecipitated with anti‐Myc antibody, followed by immunoblotting with anti‐Flag antibody (A) or immunoprecipitated with anti‐Flag antibody, followed by immunoblotting with anti‐Myc antibody (B). (C, D) Cell lysates of H1299 (C) and A549 (D) were immunoprecipitated with anti‐USP35 antibody or IgG antibody, followed by immunoblotting with anti‐RRBP1 antibody. (E) H1299 and A549 cells were stained with anti‐USP35 and anti‐RRBP1 antibodies, followed by corresponding TRITC‐conjugated and FITC‐conjugated secondary antibodies staining. Merged image (yellow) shows the overlap of USP35 and RRBP1 staining. Images were captured respectively via confocal microscopy (H1299) and fluorescence microscopy (A549). (F, G) H1299 cells were costained anti‐USP35 or anti‐RRBP1 antibodies with the ER‐resident protein calnexin, followed by corresponding TRITC‐conjugated and FITC‐conjugated secondary antibodies staining. Merged images (yellow) showed the overlapped areas of USP35 or RRBP1 with calnexin. Images were captured respectively via confocal microscopy. Scale bars indicate 50 µm (E–G) and 10 µm in the inserts (E–G). All results are representatives of three independent experiments.

To further support that RRBP1 is a direct target of USP35, Co‐IP analysis was performed to test the interaction between USP35 and RRBP1. HEK293T cells were cotransfected with Myc‐tagged USP35 and Flag‐tagged RRBP1 or Flag‐tagged empty vector, respectively. The proteasome inhibitor MG132 can result in the accumulation of USP35 and RRBP1 in cells. To facilitate the detection of their interactions, the cells of each group were treated with 10 µm MG132 for 6 h. Immunoprecipitation and subsequent immunoblot analysis showed that USP35 could interact with RRBP1 in a reciprocal fashion (Fig. 3A,B). In addition, such interaction was also confirmed at the endogenous level in H1299, A549, and Anip973 cells, indicating a physical interaction between them (Fig. 3C,D, Fig. S3). Subsequently, the localization of the two proteins was detected by immunofluorescence. Confocal and fluorescence microscopy analyses demonstrated that RRBP1 (green) was predominantly expressed in perinuclear region of the cells, while USP35 (red) was detected throughout the cell with a slightly augmented expression in the cytosol. The colocalization (yellow) of USP35 and RRBP1 is mainly at perinuclear regions in H1299 and A549 cells (Fig. 3E). Moreover, to further assess whether the interaction of USP35 and RRBP1 occurs at the level of ER, we costained USP35 or RRBP1 with the ER‐resident protein calnexin in H1299 cells. As indicated in Fig. 3F,G, both the fluorescences of USP35 and RRBP1 showed a clear overlap with the fluorescence of calnexin, implying that USP35 and RRBP1 interact at ER. Taken together, these data indicated that USP35 could directly interact with RRBP1.

### 3.4 USP35 alleviates ERS‐induced cell apoptosis

**Fig. 4:**
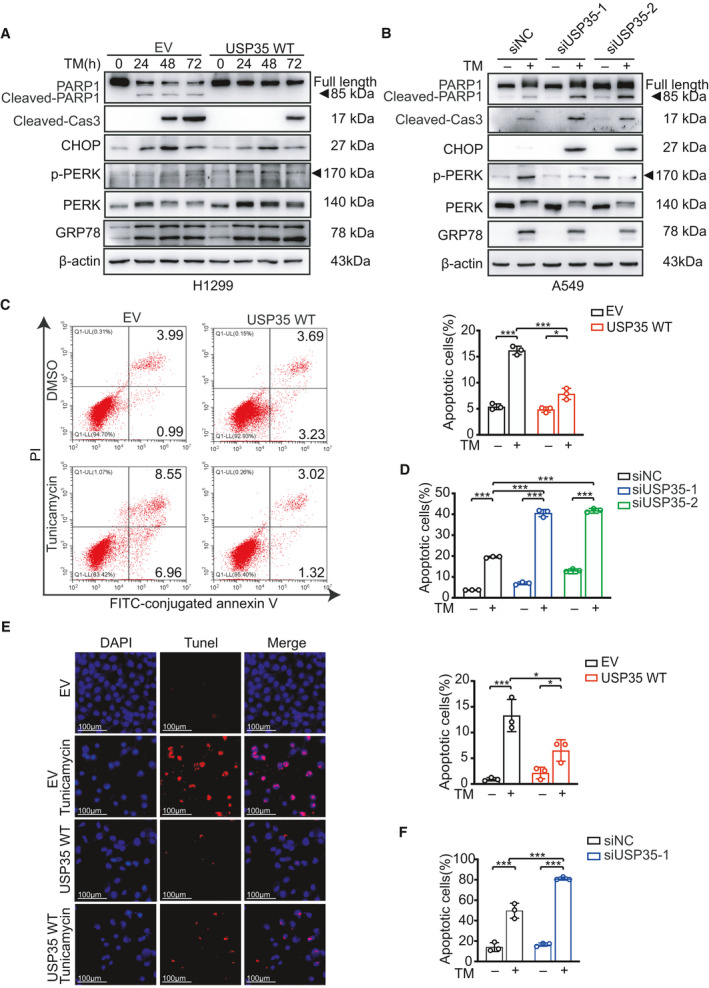
USP35 overexpression inhibits TM‐induced cell apoptosis. (A) H1299 cells with stable overexpression of USP35 (USP35 WT) and their control (EV) cells were treated with 2 µm TM for 0, 24, 48, and 72 h. The indicated proteins were detected by western blot. (B) A549 cells transfected with USP35‐specific siRNAs (siUSP35‐1 and siUSP35‐2) and scramble siRNA (siNC) were treated with or without 2 µm TM for 48 h. The indicated proteins were detected by western blot. (C, D) USP35 WT and its control H1299 cells (C) and siUSP35‐1, siUSP35‐2, and siNC A549 cells (D) were treated with or without 2 µm TM for 48 h. The cells were subsequently stained with Annexin V‐FITC and propidium iodide (PI) and analyzed by flow cytometry. Quantitative analyses were shown in the graphs. (E, F) USP35 WT and its control H1299 cells (E), siUSP35‐1 and siNC A549 cells (F) were treated with or without 2 µm TM for 48 h. The apoptotic cells were detected using TUNEL staining. Quantitative analyses were shown in the graphs. Scale bars indicate 50 µm (E). All data are presented by mean ± SD. *P < 0.05, ***P < 0.001 based on the Student *t*‐test. All results are representatives of three independent experiments.

RRBP1 has been reported to be associated with ER stress (ERS)‐induced apoptosis. We therefore speculated that USP35 may also involve in such process in NSCLC cells. TM, a well‐characterized ER stress‐inducing agent that inhibits protein glycosylation, was used in our experiments. The indicated cells were treated with different concentrations (0.5, 1, and 2 µm) of TM for different time (24, 48, and 72 h) to model the ERS condition. Phosphor‐protein kinase R‐like endoplasmic reticulum kinase (PERK) is one of the three arms (PERK, IRE1‐α, and ATF6) of UPR sensors [39]. Under minor reversible ERS conditions, ER chaperone, the 78‐kDa glucose‐regulated protein (GRP78, also known as BiP or HSPA5) is induced and dissociated from UPR arms to facilitate protein folding and PERK is dimerized and phosphorylated as an active form to phosphorylate eukaryotic translation initiation factor 2A (eIF2α), resulting in translation attenuation as an adaptive response [40]. Under persistent irreversible ERS, PERK‐ATF4‐CHOP signaling is activated to initiate apoptotic process as a result of adaptive failure [41]. In line with our hypothesis, GRP78 and phosphorylation of PERK were induced by TM treatment in a time‐ and dose‐dependent manner, and phosphor‐PERK was further increased in USP35 overexpression group, indicating the promoting effect of USP35 on UPR activation upon ER stress (Fig. 4A, Fig. S4A,B). Concomitantly, the pro‐apoptotic mediator, C/EBP homologous protein (CHOP) along with other two apoptosis marker, cleaved caspase‐3 and cleaved PARP1 were also triggered by TM treatment. However, instead of augmentation of these apoptotic‐associated molecules upon ERS induction, USP35 overexpression actually suppressed the expression of these molecules, implying that USP35 was more prone to facilitate an anti‐apoptotic/prosurvival stress‐relieving process rather than contribute to an apoptotic result (Fig. 4A, Fig. S4A,B). Consistently, USP35 silencing boosted the TM‐induced expression of CHOP, cleaved caspase‐3, and cleaved PARP1 accompanied with restrained increase of GRP78 and phosphor‐PERK triggered by TM in A549 cells, further confirming our belief about the ERS alleviating roles of USP35 (Fig. 4B, Fig. S5B). It was worth noting that unlike the widely accepted classical PERK‐ATF4‐CHOP signaling pathway where phosphor‐PERK induces expression of CHOP in response to ER stress, we found a contradictory relationship between phosphor‐PERK and CHOP expression in the context of aberrant expression of USP35, suggesting the existence of other molecules interfering with this regulatory chain. As it has been reported that phosphor‐PERK could trigger phosphorylation of NF‐E2‐related factor 2 (Nrf2) and the latter inhibited CHOP expression through antagonizing the recruitment of ATF4 to CHOP promoter [42], it is possible that similar negative modulators or signaling which might be regulated in some ways by USP35 are implicated here. However, the exact mechanisms need to be further explored.

Then, the effect of USP35 on TM‐induced cell apoptosis was further evaluated by flow cytometry analysis and Tunnel assay. Flow cytometric analysis of FITC‐conjugated Annexin V and propidium iodide (PI) staining showed that H1299‐EV cells exhibited a higher apoptotic rate with TM treatment compared to H1299‐USP35 cells with the same treatment (Fig. 4C). In contrast, TM treatment resulted in more cell apoptosis in A549‐siUSP35 cells than that in the control cells (Fig. 4D, Fig. S5C). The knockdown efficiency of siRNAs targeting USP35 or RRBP1 was confirmed by western blot (Fig. S5A). In parallel, such effect of USP35 on TM‐induced cell apoptosis was also confirmed by TUNEL assay (Fig. 4E,F, Fig. S5D). Altogether, these data suggested that USP35 overexpression mitigated ER stress inducer‐triggered cell apoptosis.

### 3.5 RRBP1 mediates the protective roles of USP35 in ERS‐induced apoptosis

**Fig. 5:**
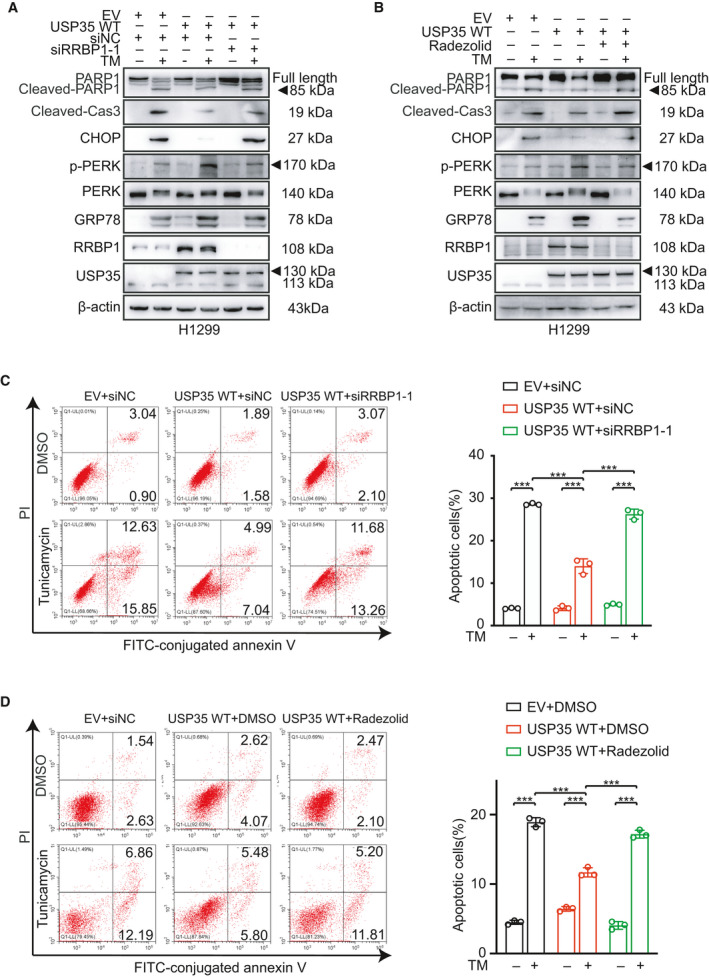
USP35 overexpression attenuates TM‐induced cell apoptosis through up‐regulating RRBP1. (A) RRBP1‐specific siRNA (siRRBP1‐1) or control siRNA was introduced into USP35 overexpression (USP35 WT) H1299 cells. Then, the cells were treated with or without 2 µm TM for 48 h. The indicated proteins were detected by western blot. (B) USP35 overexpressed (USP35 WT) H1299 cells were treated with Radezolid (RRBP1 inhibitor, 10 µm) or TM (2 µm) alone or in combination for 48 h. The indicated proteins were detected by western blot. (C, D) The apoptotic rates of aforementioned H1299 cells were detected by flow cytometry. Quantitative analyses were shown in the graphs. All data are presented by mean ± SD. ***P < 0.001 based on the Student *t*‐test. All results are representatives of three independent experiments.

**Fig. 6:**
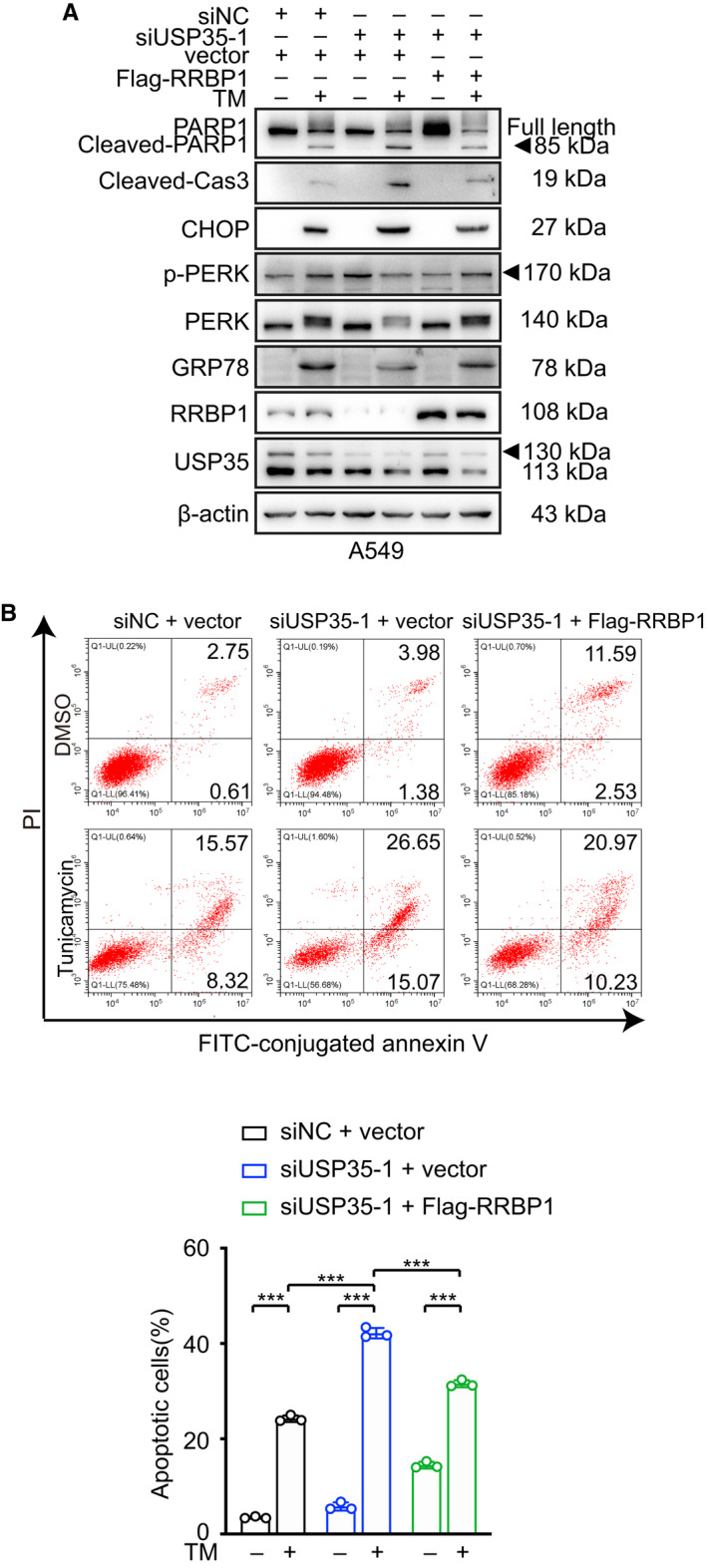
USP35 silencing boosts TM‐induced cell apoptosis through down‐regulating RRBP1. (A) Flag‐RRBP1 expression plasmid or control plasmid was introduced into USP35 silenced (siUSP35‐1) A549 cells. Then, the cells were treated with or without 2 µm TM for 48 h. The indicated proteins were detected by western blot. (B) The apoptotic rates of aforementioned A549 cells were detected by flow cytometry. Quantitative analyses were shown in the graphs. All data are presented by mean ± SD. ***P < 0.001 based on the Student *t*‐test. All results are representatives of three independent experiments.

To further explore whether USP35 exerted its anti‐apoptotic roles through stabilizing RRBP1, we silenced RRBP1 expression by RNA interference or by Radezolid, a potential RRBP1 inhibitor [43] in USP35‐overexpressed cells or overexpressed RRBP1 in USP35‐knockdown cells in the presence of TM. Western blot results showed that upon TM induction, both genetic knockdown and pharmacological inhibition of RRBP1 restored expression of CHOP, cleaved caspase‐3, and cleaved PARP1 in USP35 overexpressed H1299 cells (Fig. 5A,B, Figs S6A,B and S9A,B). Accordingly, ectopic expression of RRBP1 in A549‐siUSP35 cells abrogated the enhancement of the mentioned proteins caused by USP35 silencing by TM treatment (Fig. 6A, Fig. S7). In keeping with this finding, RRBP1 knockdown increased the sensitivity of H1299‐USP35 cells to TM treatment as indicated by elevated cell death from 13.99 ± 1.73 to 26.26 ± 1.18 (Fig. 5C). Pharmacological blockade of RRBP1 increased the H1299‐USP35 cell death from 11.69 ± 0.66 to 17.18 ± 0.57 on TM treatment (Fig. 5D). Meanwhile, RRBP1 re‐introduction in A549‐siUSP35 cells decreased TM sensitivity as evidenced by reduced cell death from 42.18 ± 1.08 to 31.57 ± 0.73 (Fig. 6B). Together, these results suggested that USP35 alleviated TM‐induced apoptosis by increasing RRBP1 expression.

### 3.6 USP35 positively correlates with the RRBP1 expression as well as the poor prognosis in lung adenocarcinoma patients

**Fig. 7:**
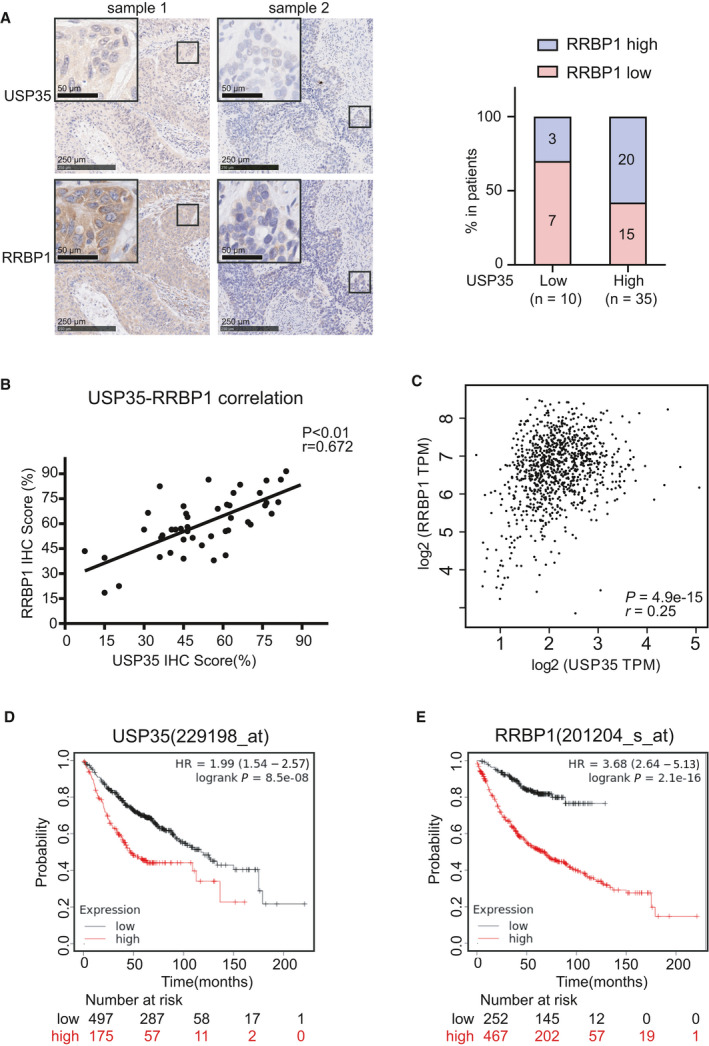
RRBP1 expression positively correlates with USP35 expression in NSCLC tissues. (A) The representative immunohistochemistry staining of USP35 and RRBP1 in NSCLC tissues were shown. The box plot indicated the relative RRBP1 level in USP35‐low and USP35‐high patients (median USP35 or RRBP1 expression was defined as cutoff point). Scale bars indicate 250 and 50 µm in the inserts. (B, C) The correlation between the expression of USP35 and RRBP1 in NSCLC tissues (*n* = 45) (B) and the correlations between the expression of USP35 and RRBP1 in NSCLC tissues in TCGA database from GEPIA (*n* = 969) (C) were presented in scatter plot. (D) The Kaplan–Meier Plotter database (229198_at) analyzed the USP35 levels in relation to the overall survival of patients with lung adenocarcinoma (Cutoff value used in analysis: 142). (E) The Kaplan–Meier Plotter database (201204_s_at) analyzed the RRBP1 levels in relation to the overall survival of patients with lung adenocarcinoma (cutoff value used in analysis: 681).

To explore clinical relevance of our findings, we then analyzed the expression of USP35 and RRBP1 in NSCLC tissues. We performed IHC using specific antibodies against USP35 and RRBP1 on 45 lung cancer tissue samples. Quantification of the immunostaining and statistical analysis showed that RRBP1 expression positively correlated with USP35 (Fig. 7A,B). The correlation between USP35 and RRBP1 protein was also supported by the GEPIA data (http://gepia.cancer‐pku.cn/) (Fig. 7C), reinforcing the notion that RRBP1 was a bona fide substrate of USP35 in NSCLC.

In addition, we analyzed relationship between USP35 and RRBP1 expression and several clinicopathological features of patients with NSCLC. As shown in Tables S5 and S6, both USP35 and RRBP1 expression levels were significantly higher in more advanced TNM stages, but were not significantly affected by sex, age, or tumor size. We further assessed the association between USP35 and RRBP1 expression and the survival of lung cancer patients using Kaplan–Meier Plotter database (http://kmplot.com) and found that high expression of USP35 or RRBP1 was associated with shorter overall survival in lung adenocarcinoma patients (Fig. 7D,E).

Moreover, as the well‐characterized ER stress‐related protein GRP78 has been reported to be closely related to the prognosis of lung adenocarcinoma patients [44], we therefore investigated the correlation of USP35 and RRBP1 with GRP78 in the public database GEPIA. Statistical analysis showed positive correlations between USP35 and GRP78 as well as RRBP1 and GRP78 in these samples (*P* = 0.0093; *P* < 0.0001) (Fig. S8A,B). Together, these data indicated that the expression of USP35 and RRBP1 served as critical prognostic predictors for NSCLC patients.

## 4 Discussion

As a newly identified DUB, USP35 has been reported to regulate cell proliferation, cell mitosis, and malignant progression [45, 46]. However, the biological roles of USP35 in tumorigenesis are vague and controversial at present. Our previous study has implied that USP35 may function as a tumor suppressor for the reason that overexpression of USP35 inhibits cell proliferation [24]. More recently, USP35 has been reported to play oncogenic roles in ovarian cancer through targeting stimulator of interferon genes (STING) pathway [25]. These conflicting data indicate that USP35 may have context‐specific functions and there is no one‐size‐fits‐all conclusion. Specific roles in different cancer types and detailed mechanisms of USP35 need to be delineated. Given that DUBs usually exert biological roles through modulating different targeting proteins, in this study, we applied global proteomic screening to identify proteins that were differentially expressed in USP35 overexpression or knockdown in H1299 cells to better understand the interactomes of USP35. To the best of our knowledge, it was the first study to globally screen the potential targets of USP35 using proteomic analysis. As a result, among the DEPs induced by USP35 aberrant expression, the expression of RRBP1 showed a significant positive correlation with the expression of USP35, raising the possibility that RRBP1 could be a putative substrate of USP35 in NSCLC.

Recently, RRBP1 has been identified as an important oncogenic factor to induce tumorigenesis and malignant progression. RRBP1 is reported to be highly expressed in multiple cancers and may serve as a potential biomarker for prognostic prediction [27, 28, 29, 32, 47, 48]. Although several studies have shown that the enhanced RRBP1 expression in tumor cells is attributed to its transcriptional and translational activation [49, 50, 51], nothing is known about whether RRBP1 can be regulated at post‐translational level.

An important implication of our study is that RRBP1 abundance could be regulated by USP35 using proteomics analysis, which was validated further with western blot in different NSCLC cells with different USP35 expression status. In the following attempts to dissect the molecular mechanisms underlying USP35‐mediated regulation of RRBP1 abundance, we found that USP35 could interact with RRBP1 physically and regulate RRBP1 stability in its DUB activity‐dependent manner. RRBP1 became more stable in USP35 overexpressing cells, whereas RRBP1 had a shorter half‐life in USP35 knockdown cells. Furthermore, the ubiquitination of RRBP1 was increased by USP35 knockdown but decreased by ectopic expression of USP35. These data confirmed a bona fide target of USP35–RRBP1, which can be deubiquitinated and stabilized at post‐translational level.

Given the critical role of RRBP1 in regulating ER stress‐induced apoptosis and hitherto confirmed interaction between USP35 and RRBP1, we further demonstrated that overexpression of USP35 protected NSCLC cells from ERS‐triggered apoptosis, while silencing USP35 exacerbated this phenotype, indicating anti‐apoptotic role of USP35 during ERS. Our study further confirmed that RRBP1 was the mediator for USP35 to exert anti‐apoptotic role upon ER stressor induction in NSCLC cells. Evading apoptosis is a crucial hallmark of cancer progression. Recently, accumulating evidence emphasizes the importance of ER stress response in apoptosis [21, 52, 53]. Tumor cells are exposed to multiple extracellular and intracellular perturbations including hypoxia, nutrient deprivation, oncogenic activation and genome instability, which can disrupt ER proteostasis and lead to the accumulation of misfolded proteins at the ER lumen (known as ER stress) followed by subsequent activation of UPR [7, 54, 55]. On one hand, activated UPR can exert its adaptive prosurvival roles to alleviate the damage and restore cellular homeostasis through specific signal transduction pathway. On the other hand, under sustained or severe ER stress, prolonged activation of the UPR can execute antisurvival function to lead to apoptotic cell death through death‐triggering pathways such as PERK‐ATF4‐CHOP signaling. Thus, fine‐tuning of the output of ER stress from the prosurvival branch to the pro‐apoptotic branch provides a promising optional for precision cancer therapy. However, to date, the modulators that regulate the output of ER stress have not been fully clarified. Our present finding indicates that USP35 expression level could affect the fate of tumor cells when stressful stimuli are presented. In consideration of the lack of *in vivo* data, in‐depth signaling explorations and better ERS models, more studies need to be conducted to further clarify the roles and molecular mechanisms of USP35 involved in ERS.

Finally, to address the clinical significance of USP35‐RRBP1 axis, we assessed the prognostic values of USP35 and RRBP1 expression and found that their high expressions were associated with shorter overall survivals. Our findings indicate that USP35‐RRBP1 axis may act as a new biomarker for outcome prediction of NSCLC patients.

## 5 Conclusions

Our study identified RRBP1 as a new substrate of USP35 and USP35 alleviated ERS‐induced cell apoptosis by stabilizing RRBP1 through the proteasome pathway. This finding suggests that USP35 is a potential player in regulating the intracellular environment to maintain homeostasis. Targeting USP35‐RRBP1 axis may provide an attractive therapeutic strategy for NSCLC patients.

## Conflict of interest

The authors declare no conflict of interest.

## Author contributions

PZ and XL designed the study. WW, MW, YX, YW, LM, LG, and XW performed the experiments and the data analysis. WW wrote the manuscript. PZ and YX edited the manuscript. All authors read and approved the final manuscript.

## Supporting information


**Fig. S1.** USP35 enhances RRBP1 expression at posttranscriptional level.
**Fig. S2.** USP35 decreases ubiquitination of RRBP1.
**Fig. S3.** USP35 interacts with RRBP1.
**Fig. S4.** USP35 overexpression alleviates ER stress induced cell apoptosis.
**Fig. S5.** USP35 knockdown exacerbates ER stress induced cell apoptosis.
**Fig. S6.** RRBP1 is responsible for the inhibitory effect of USP35 on Tunicamycin (TM)‐induced cell apoptosis.
**Fig. S7.** RRBP1 overexpression inhibits the promotive effect of USP35 silencing on Tunicamycin (TM)‐induced cell apoptosis.
**Fig. S8.** GPR78 expression is positively correlated with USP35 and RRBP1 expression in NSCLC tissues.
**Fig. S9.** USP35 overexpression enhances Tunicamycin (TM)‐induced phosphorylation of PERK through up‐regulating RRBP1.
**Table S1.** The Types, Dilutions and Sources of Antibodies Used for western Blot and Immunohistochemical Analysis.
**Table S2.** Differential expression proteins (DEPs) induced by USP35 overexpression.
**Table S3.** Differential expression proteins (DEPs) induced by USP35 silencing.
**Table S4.** The 26 overlapping proteins between USP35‐upregulated proteins and shUSP35‐downregulated proteins.
**Table S5.** Correlation between expression levels of USP35 and the clinical features.
**Table S6.** Correlation between expression levels of RRBP1 and the clinical features.

## Data Availability

The data supporting this study are available from the corresponding authors on reasonable request. The prognostic significance of the USP35 and RRBP1 expression in NSCLC were evaluated using the Kaplan–Meier Plotter (www.kmplot.com). The correlation between USP35 and GRP78 expression as well as RRBP1 and GPR78 expression in NSCLC tissues was assessed using Gene Expression Profiling Interactive Analysis (GEPIA2; http://gepia2.cancer‐pku.cn/#index).
