# Supplementary material for: USP35 mitigates endoplasmic reticulum stress‐induced apoptosis by stabilizing RRBP1 in non‐small cell lung cancer
Source: Mol Oncol. 2021 Oct 18;16(7):1572–90. doi: 10.1002/1878-0261.13112 (PMC8978513; doi:10.1002/1878-0261.13112)
Supplement: Supplementary file 1 — Fig. S1. USP35 enhances RRBP1 expression at posttranscriptional level. Fig. S2. USP35 decreases ubiquitination of RRBP1. Fig. S3. USP35 interacts with RRBP1. Fig. S4. USP35 overexpression alleviates ER stress induced cell apoptosis. Fig. S5. USP35 knockdown exacerbates ER stress induced cell apoptosis. Fig. S6. RRBP1 is responsible for the inhibitory effect of USP35 on Tunicamycin (TM)‐induced cell apoptosis. Fig. S7. RRBP1 overexpression inhibits the promotive effect of USP35 silencing on Tunicamycin (TM)‐induced cell apoptosis. Fig. S8. GPR78 expression is positively correlated with USP35 and RRBP1 expression in NSCLC tissues. Fig. S9. USP35 overexpression enhances Tunicamycin (TM)‐induced phosphorylation of PERK through up‐regulating RRBP1. Table S1. The Types, Dilutions and Sources of Antibodies Used for western Blot and Immunohistochemical Analysis. Table S2. Differential expression proteins (DEPs) induced by USP35 overexpression. Table S3. Differential expression proteins (DEPs) induced by USP35 silencing. Table S4. The 26 overlapping proteins between USP35‐upregulated proteins and shUSP35‐downregulated proteins. Table S5. Correlation between expression levels of USP35 and the clinical features. Table S6. Correlation between expression levels of RRBP1 and the clinical features. [file MOL2-16-1572-s001.pdf]

Supplementary materials

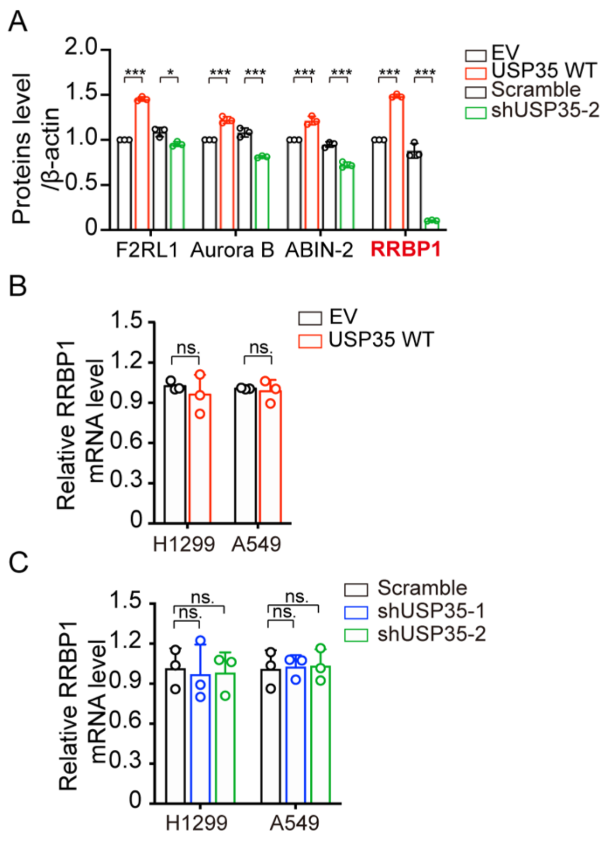

Fig. S1. USP35 enhances RRBP1 expression at posttranscriptional level.

(A) Quantitative analysis of the indicated DEPs was confirmed by western blot. (B) Expression of RRBP1 was detected by qRT-PCR in USP35 overexpression (USP35 WT) H1299 and A549 cells. (C) Expression of *RRBP1* was detected by qRT-PCR in shRNAs mediated USP35 knockdown H1299 and A549 cells. All data are presented by mean ± SD. $^{*}P<0.05$, $^{***}P<0.001$ based on the Student *t*-test. All results are representatives of three independent experiments.

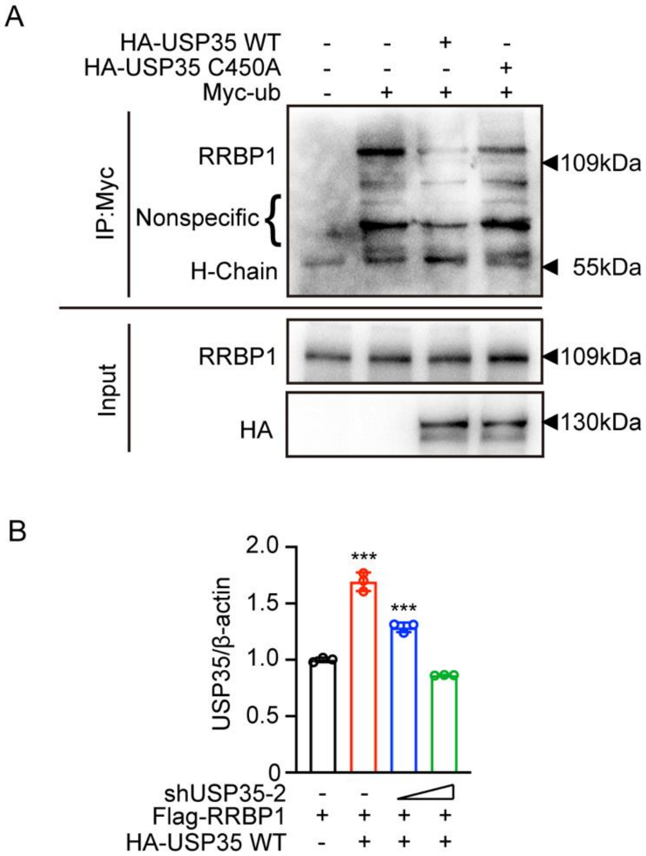

Fig. S2. USP35 decreases ubiquitination of RRBP1.

(A) H1299 cells were transfected or co-transfected with Myc-Ub alone or along with HA-USP35 WT or HA-USP35 C450A. Cell lysates were immunoprecipitated with anti-Myc antibody, followed by immunoblotting with an anti-RRBP1 antibody. The results are representatives of three independent experiments. (B) Quantitative analyses of USP35 protein detected by western blot in the indicated PC9 cells transfected or co-transfected with Flag-RRBP1 alone or along with HA-USP35 WT or in combination with HA-USP35 WT and 2 μg or 4 μg shUSP35-2. All data are presented by mean ± SD. ****P<0.001* based on the Student *t*-test.

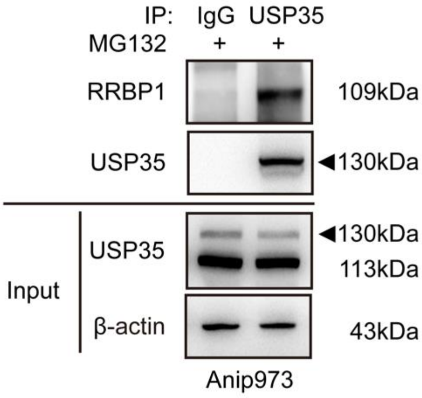

Fig. S3. USP35 interacts with RRBP1.

Cell lysates of Anip973 were immunoprecipitated with anti-USP35 antibody or IgG antibody, followed by immunoblotting with anti-RRBP1 antibody. The results are representatives of three independent experiments.

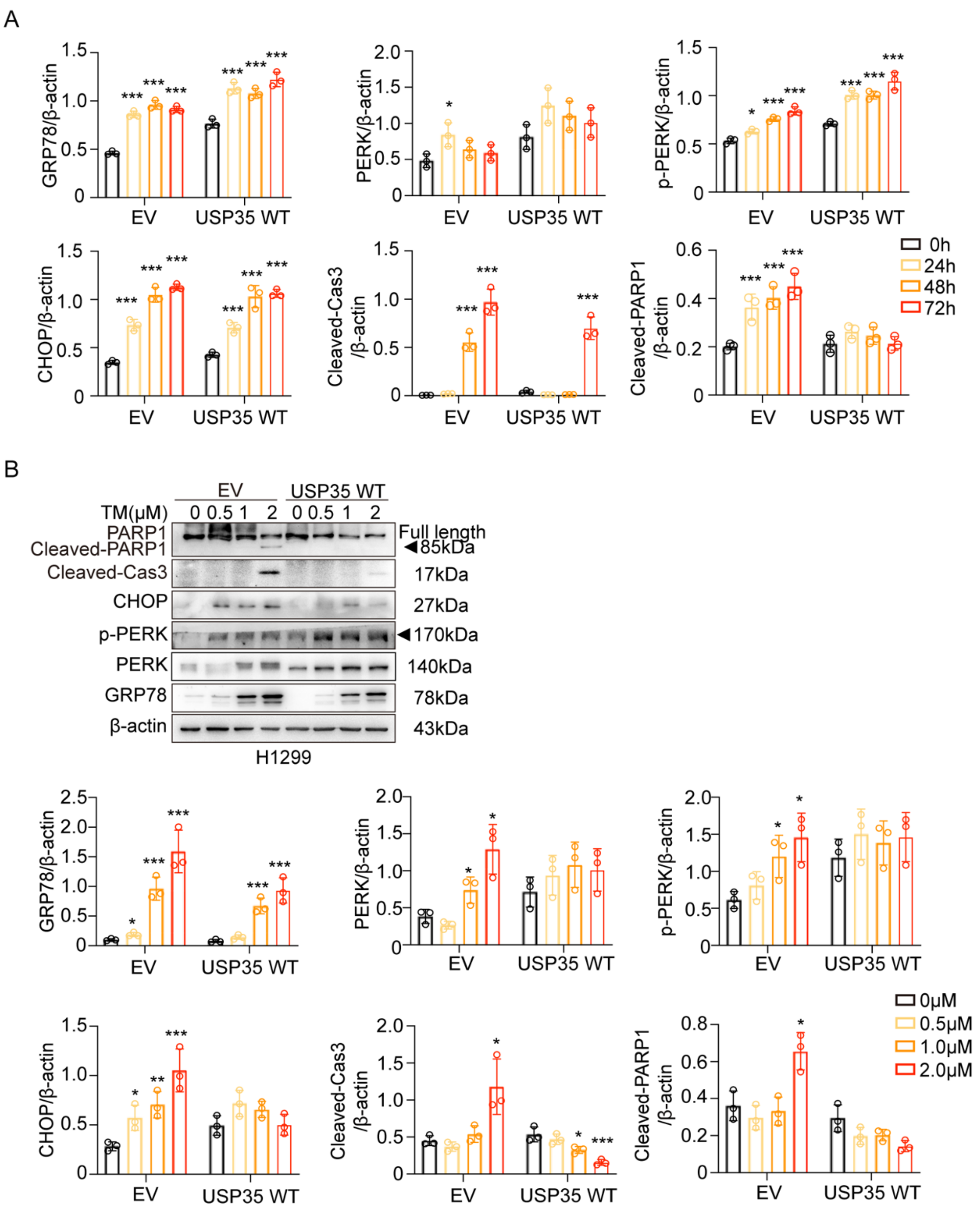

Fig. S4. USP35 overexpression alleviates ER stress induced cell apoptosis.

(A) Quantitative analyses of the indicated proteins detected by western blot in H1299 cells with stable overexpression of USP35 (USP35 WT) and their control (EV) cells treated with 2 μM TM for 0 h, 24 h, 48 h and 72 h. (B) H1299 cells with stable overexpression of USP35 (USP35 WT) and their control (EV) cells were treated with 0 μM, 0.5 μM, 1 μM and 2 μM TM for 48 h. The indicated proteins were detected by western blot. Quantitative analyses were shown in the graphs.

All data are presented by mean ± SD. *$P<0.05$, ***$P<0.001$ based on the Student *t*-test. All results are representatives of three independent experiments.

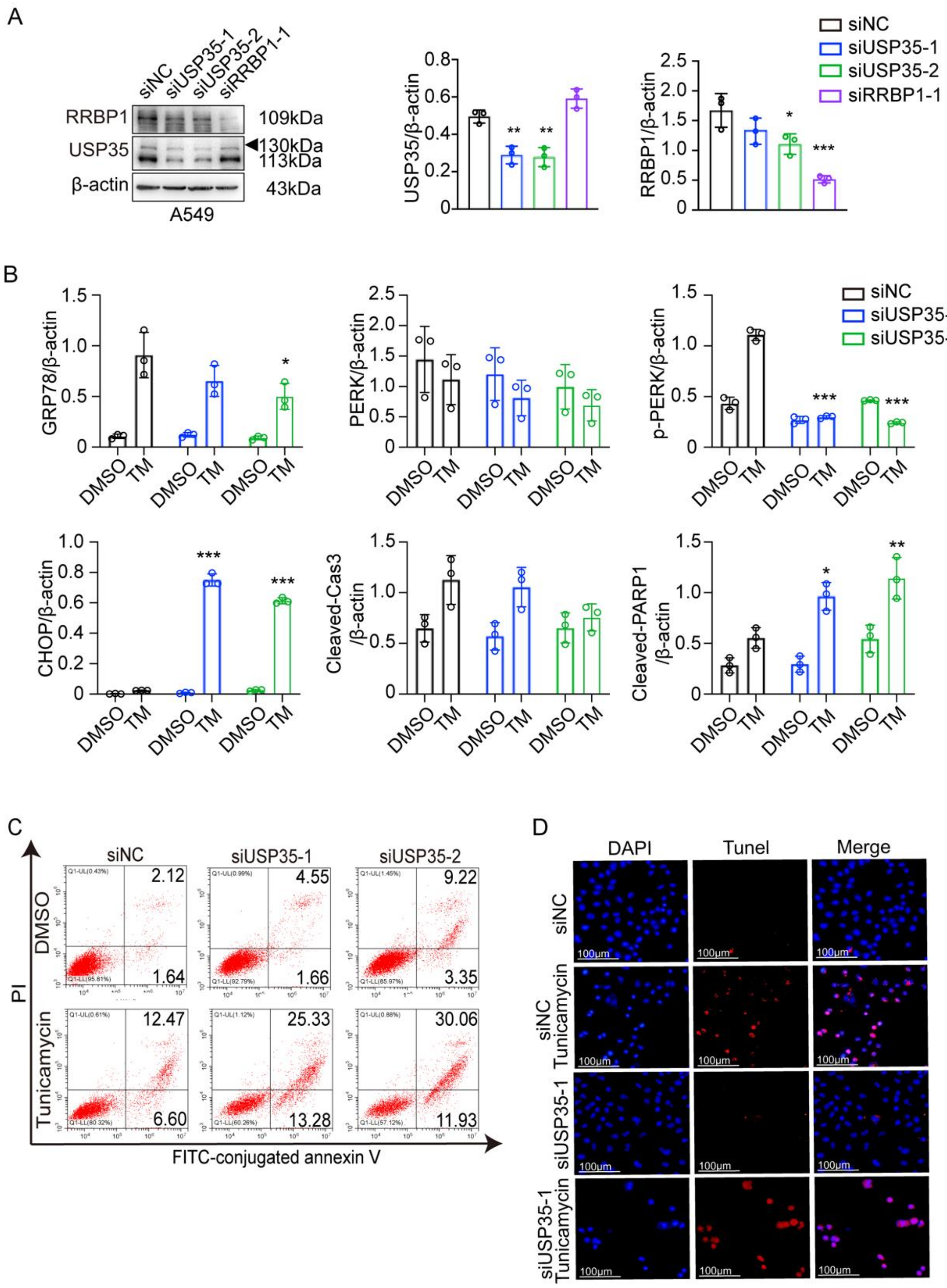

Fig. S5. USP35 knockdown exacerbates ER stress induced cell apoptosis.

(A) The knockdown efficiency of siRNAs specifically targeting USP35 or RRBP1 was verified by western blot in A549 cells. Quantitative analyses were shown in the graphs. (B) Quantitative analyses were presented for the indicated proteins detected

by western blot in A549 cells transfected with USP35 specific siRNAs (siUSP35-1 and siUSP35-2) and scramble siRNA (siNC) treated with or without 2 μM TM for 48 h. (C) SiUSP35-1, siUSP35-2 and siNC A549 cells were treated with or without 2 μM TM for 48 h. The cells were subsequently stained with Annexin V-FITC and propidium iodide (PI) and analyzed by flow cytometry. (D) SiUSP35-1 and siNC A549 cells were treated with or without 2 μM TM for 48 h. The apoptotic cells were detected using TUNEL staining. Scale bars indicate 100 μm (D). All data are presented by mean ± SD. *$P<0.05$, *$P<0.01$, ***$P<0.001$ based on the Student *t*-test. All results are representatives of three independent experiments.

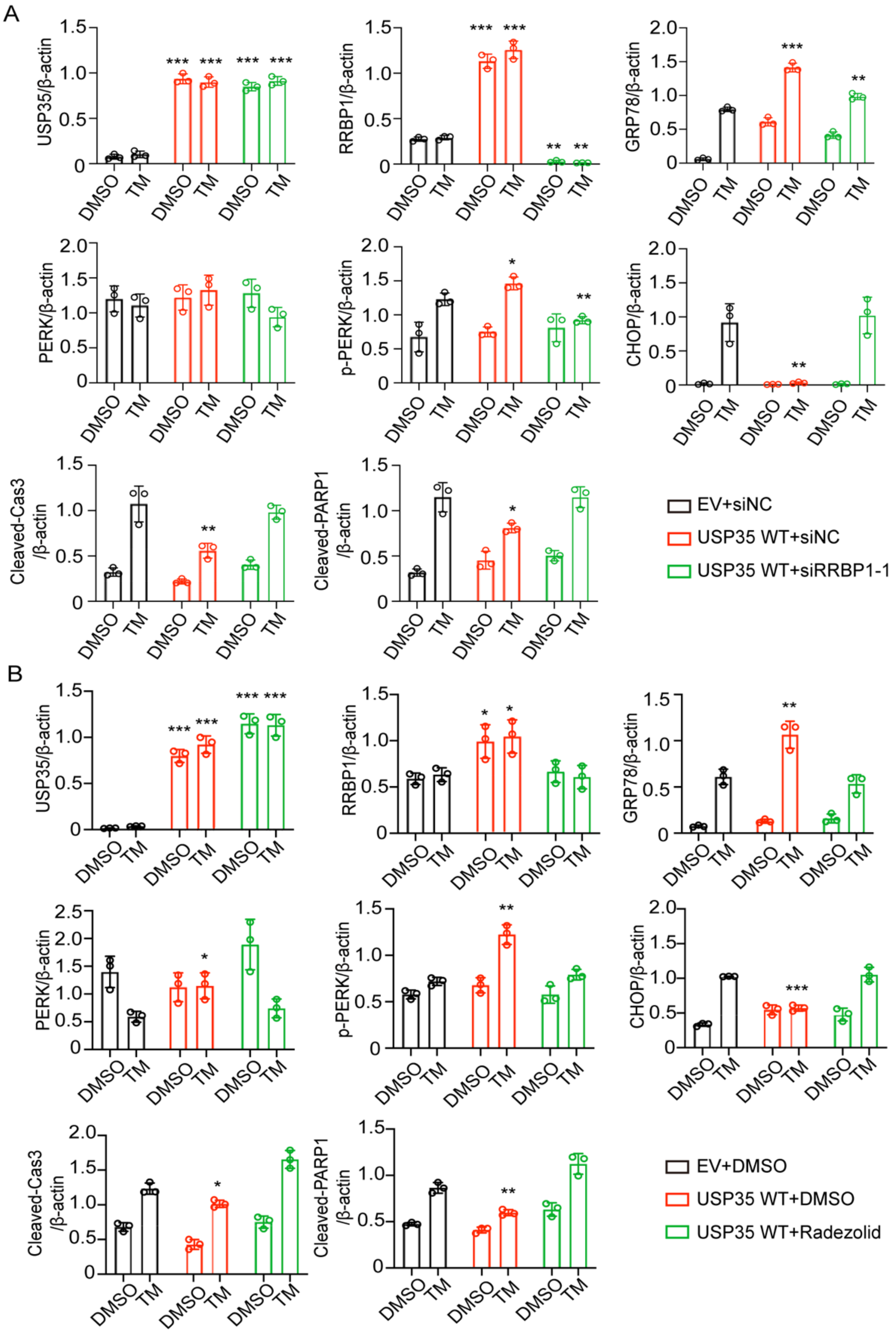

Fig. S6. RRBP1 is responsible for the inhibitory effect of USP35 on Tunicamycin (TM)-induced cell apoptosis.

(A and B**)** Quantitative analyses were presented for the indicated proteins detected by western blot in the indicated H1299 cells treated with or without 2 μM TM (A) or in

combination with Radezolid (B) for 48h. All data are presented by mean ± SD. *$P<0.05$, **$P<0.01$, ***$P<0.001$ based on the Student *t*-test.

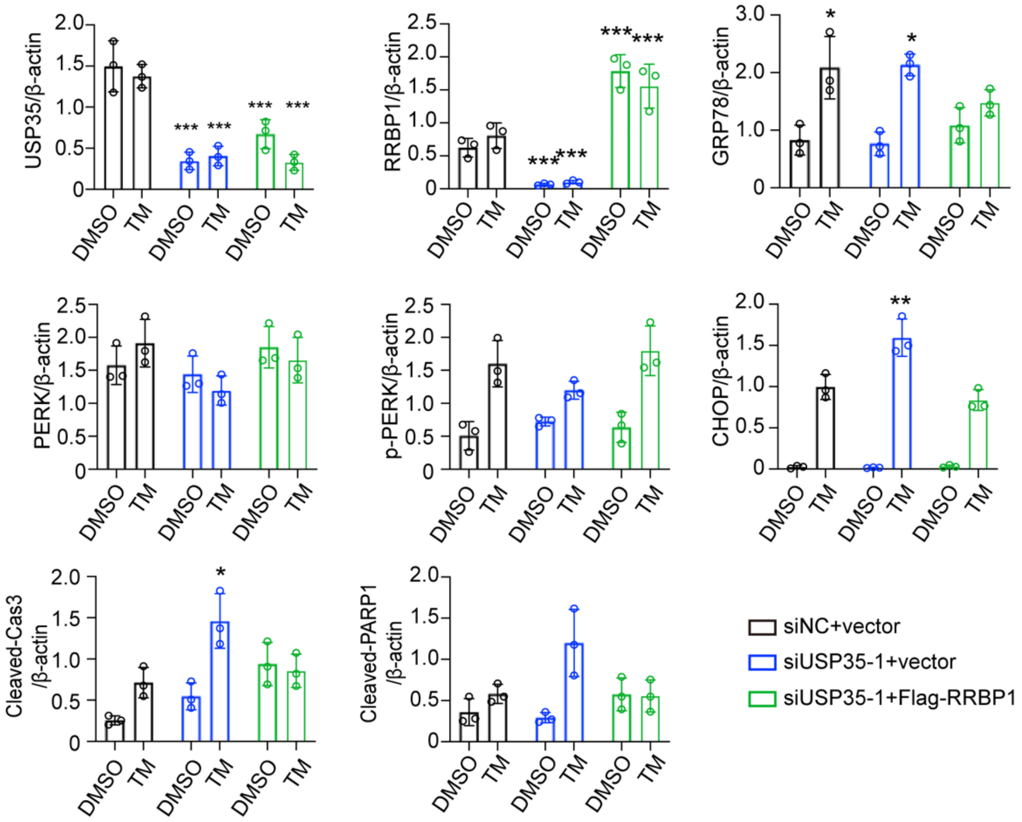

Fig.S7. RRBP1 overexpression inhibits the promotive effect of USP35 silencing on Tunicamycin (TM)-induced cell apoptosis.

Quantitative analyses were shown for the indicated proteins detected by western blot in the indicated A549 cells treated with or without 2 μM TM for 48 h. All data are presented by mean ± SD. **P<0.05*, ***P<0.01*, ****P<0.001* based on the Student *t*-test.

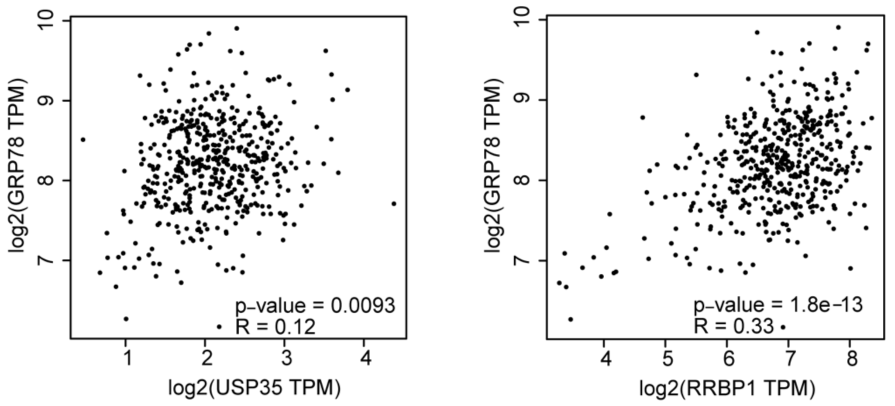

Fig.S8. GPR78 expression is positively correlated with USP35 and RRBP1 expression in NSCLC tissues.

(A) The correlation between the expression of GPR78 and USP35 in NSCLC tissues from GEPIA was presented in scatter plot. (B) The correlation between the expression of GPR78 and RRBP1 in NSCLC tissues from GEPIA was presented in scatter plot.

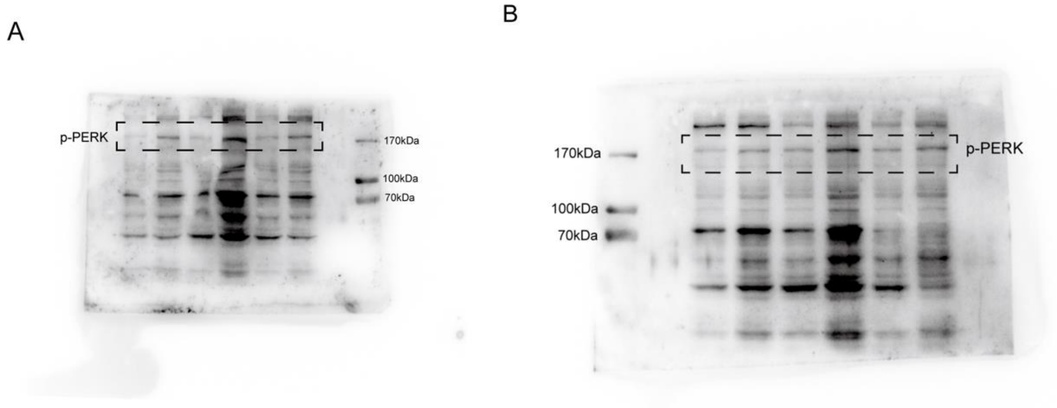

Fig.S9. USP35 overexpression enhances Tunicamycin (TM)-induced phosphorylation of PERK through up-regulating RRBP1.

(A) The uncrossed whole blots of phosphor-PERK (p-PERK) in Fig.5A. (B) The uncrossed whole blots of phosphor-PERK (p-PERK) in Fig.5B.

Supplementary Table 1 The Types, Dilutions and Sources of Antibodies Used for Western Blotting and Immunohistochemical Analysis**.**

**Antibody**	**Working dilution**	**Working dilution**	**Species**	**Source -Cat. Number**
	**Western blotting**	**IHC**		
USP35	1:1000	—	Rabbit polyclonal	Abcam (Cat. No.ab86791)
USP35	—	1:50	Rabbit polyclonal	Abcam (Cat. No.ab128592)
RRBP1	1:3000	1:100	Rabbit polyclonal	Proteintech (Cat. No. 22015-1-AP)
GRP78	1:2000	—	Rabbit monoclonal	Cell Signaling Technology (Cat. No. 3177)
PERK	1:1000	—	Rabbit monoclonal	Cell Signaling Technology (Cat. No. 5683)
Phospho-PERK (Thr980)	1:1000	—	Rabbit monoclonal	Cell Signaling Technology (Cat. No. 3179)
CHOP	1:1000	—	Mouse monoclonal	Cell Signaling Technology (Cat. No. 2895)
Cleaved-Caspase3	1:1000	—	Rabbit monoclonal	Cell Signaling Technology (Cat. No. 9661)
PARP1	1:2000	—	Rabbit polyclonal	Proteintech (Cat. No. 13371-1-AP)
Myc-tag	1:2000	—	Mouse monoclonal	Cell Signaling Technology (Cat. No. 2276)
Flag-tag	1:2000	—	Mouse monoclonal	Sigma (Cat. No.F1804)
HA-tag	1:2000	—	Mouse monoclonal	Sigma (Cat. No. H3663)
ABIN-2	1:1000	—	Mouse monoclonal	Abcam (Cat. No. ab205925)
Aurora B	1:1000	—	Mouse monoclonal	Cell Signaling Technology (Cat. No.3490)
F2RL1	1:1000		Rabbit monoclonal	Cell Signaling Technology (Cat. No.6976)
β-Actin	1:3000	—	fpolyclonal	Cell Signaling Technology (Cat. No. ab64659)

supplementary table2

Differential expressed proteins by iTARQ coupled LC-MS/MS analysis from H1299-USP35 vs H1299-EV

Uniprot Accession	Gene Symol	Fold Change	P value
A0A140VK08		4.17594	1.47E-27
V9HW43	HEL-S-102	3.94118	1.74E-25
A0A1B0GTY7	TMEM191C	3.88908	5.09E-25
R4GMP5	DLC1	3.11039	7.03E-18
Q9NZ23	YA61	3.02576	4.37E-17
P80723	BASP1	2.93243	3.30E-16
Q96HR9	REEP6	2.87556	1.14E-15
P35527	KRT9	2.70968	4.20E-14
Q96FN9	DTD2	2.61961	2.99E-13
A0A024R8C7	C9orf74	2.56085	1.07E-12
P05362	ICAM1	2.52454	2.37E-12
Q8NFZ5	TNIP2	2.5133	3.02E-12
H6VRG1	KRT1	2.47441	7.04E-12
K7WVJ5	COX2	2.4232	2.14E-11
P02533	KRT14	2.41033	2.83E-11
O75197	LRP5	2.35912	8.58E-11
Q96GD4		2.33068	1.59E-10
A0A024R407	MAP2	2.21694	1.83E-09
P25942	CD40	2.17379	4.59E-09
P28065	PSMB9	2.15042	7.54E-09
A4D126	ISPD	2.12851	1.20E-08
A0A024R4C5	ARL4C	2.10286	2.06E-08
C9JJQ8	TUBA4A	2.07287	3.88E-08
Q7Z406	MYH14	1.98886	2.24E-07
Q04695	KRT17	1.98764	2.30E-07
Q16667	CDKN3	1.94775	5.23E-07
Q15323	KRT31	1.94333	5.72E-07
Q15582	TGFBI	1.93478	6.82E-07
A0A140VK16	DPEP3	1.93052	7.44E-07
P08779	KRT16	1.92766	7.89E-07
P60880	SNAP25	1.89841	1.43E-06
P04259	KRT6B	1.88439	1.90E-06
Q00978	IRF9	1.88203	1.99E-06
Q9HC29	NOD2	1.87753	2.18E-06
P14324	FDPS	1.86903	2.59E-06
Q2TAA2	IAH1	1.8589	3.18E-06
Q16762	TST	1.85203	3.65E-06
Q96BZ9	TBC1D20	1.84953	3.83E-06
Q96RY7	IFT140	1.84665	4.06E-06
P30536	TSPO	1.84106	4.54E-06
Q6PID6	TTC33	1.82968	5.70E-06
P13647	KRT5	1.81537	7.57E-06
P84022	SMAD3	1.78599	1.35E-05
Q9NWW7	C2orf42	1.78356	1.42E-05
A0A024RDB4	HNRPD	1.77893	1.55E-05
Q96EA4	SPDL1	1.77855	1.56E-05
P01023	A2M	1.75643	2.41E-05
O95359	TACC2	1.75641	2.41E-05
A0A024R6A0	ARG2	1.74413	3.06E-05
A0A024R338	SFMBT1	1.73833	3.42E-05
Q96AB3	ISOC2	1.72934	4.06E-05
Q96H79	ZC3HAV1L	1.71814	5.04E-05
A0A0S2Z428	KRT6A	1.70715	6.21E-05
I1U8N0	HLA-A	1.70651	6.29E-05
P50454	SERPINH1	1.69888	7.27E-05
A0A024R3E3	APOA1	1.69747	7.47E-05
O00506	STK25	1.69651	7.60E-05
Q59G84		1.68931	8.71E-05
Q9NPH2	ISYNA1	1.67879	0.000106219
Q6ZUY8		1.67766	0.000108499
P02792	FTL	1.67427	0.000115629
O75521	ECI2	1.67301	0.000118395

(continued on next page)

supplementary table2 (continued)

Uniprot Accession	Gene Symol	Fold Change	P value
B2R4F3		1.67241	0.000119734
A0A024R1Y7	LGP1	1.66586	0.000135349
Q86Z14	KLB	1.66348	0.0001415
Q8TBP6	SLC25A40	1.64988	0.000182233
O14933	UBE2L6	1.64879	0.000185953
Q9H1E3	NUCKS1	1.64856	0.000186747
Q71UF1	ACO2	1.64668	0.000193363
A0A024RAD7	STEAP3	1.64181	0.000211578
Q14681	KCTD2	1.63932	0.000221526
Q14015		1.63923	0.000221894
B3KQS9		1.63636	0.000233943
A1A5C5	RRBP1	1.62918	0.000266939
Q13480	GAB1	1.62865	0.000269546
A0A0A0MSV9	TAPBP	1.62485	0.000288976
Q07021	C1QBP	1.62417	0.000292594
B2RCC2		1.6241	0.000292969
Q03135	CAV1	1.62331	0.000297233
A0A160YHU8	HLA-B	1.61259	0.000361383
P05787	KRT8	1.61077	0.000373531
A0A140VKA0	CALD1	1.60888	0.000386564
Q86SQ4	ADGRG6	1.60698	0.000400112
B2RAF2		1.59651	0.000483413
Q9MY63	HLA-Cw	1.5894	0.000549301
P17301	ITGA2	1.5888	0.000555243
Q6JQN1	ACAD10	1.58722	0.000571187
A0A024R248	C9orf89	1.58629	0.000580777
A0A087WYL5	SEZ6L2	1.58585	0.000585369
E7D7X9		1.58506	0.000593702
Q08999	RBL2	1.58171	0.000630325
Q53TN4	CYBRD1	1.56884	0.000792388
Q53G16		1.56606	0.000832335
Q15543	TAF13	1.56157	0.000900981
A2A274	ACO2	1.55952	0.000934107
H7C3I1	ST13	1.55764	0.000965516
Q53F53		1.55462	0.00101811
Q9UBG0	MRC2	1.54963	0.0011111
P54802	NAGLU	1.54672	0.00116904
A0A024R644	CLN5	1.54407	0.00122432
Q15118	PDK1	1.54302	0.00124692
Q7L5N7	LPCAT2	1.53516	0.00142923
Q9P1F3	ABRACL	1.53496	0.00143418
A8K4A8		1.52589	0.00167716
Q9UK22	FBXO2	1.52497	0.0017039
H9S5Y2	COX2	1.51914	0.00188309
A8K2B9		1.51696	0.00195462
Q9UGC6	RGS17	1.51494	0.00202321
B0QYU2	CXorf39	1.51111	0.00215964
Q5SZL2	CEP85L	1.50843	0.00226029
Q96IK0	TMEM101	1.50349	0.00245766
A0A024RAF7	ECE1	1.50342	0.00246057
Q04721	NOTCH2	1.49654	0.00276336
Q13509	TUBB3	1.49512	0.00283013
H3BRJ5		1.49369	0.00289892
B3KN29		1.49349	0.00290867
A6NFQ7	DPRX	1.49154	0.00300536
P04066	FUCA1	1.49045	0.00306073
A0A1C7CYX9	DPYSL2	1.49026	0.00307048
Q16822	PCK2	1.48581	0.00330749
A0A024R2Y6	SNRK	1.48506	0.00334912
P24844	MYL9	1.48361	0.003431
P32121	ARRB2	1.48343	0.0034413
A0A087WX23	PEG10	1.48219	0.00351303

(continued on next page)

supplementary table2 (continued)

Uniprot Accession	Gene Symol	Fold Change	P value
B7Z1N4		1.48195	0.00352708
A2VBB7	HLA-B	1.48119	0.00357192
D3DQU2	TPP1	1.47867	0.0037245
Q06481	APLP2	1.4786	0.00372882
P14384	CPM	1.47569	0.00391292
A0A024R7D2	SLC44A2	1.47511	0.00395063
Q8NFH9		1.4729	0.0040975
B4DEE8		1.47106	0.00422372
L7N2F9		1.47004	0.00429527
Q9NZN3	EHD3	1.46628	0.00456907
A0A024R3Z5	LANCL1	1.46512	0.00465682
Q13232	NME3	1.46341	0.00478909
P78310	CXADR	1.46059	0.00501503
Q96IR7	HPDL	1.45695	0.00532161
V9HW87	HEL-S-299	1.4566	0.00535201
A0A024RDL9	PSPH	1.45622	0.00538518
Q9Y654	CBX1	1.45324	0.00565224
Q9C0B7	TANGO6	1.45291	0.00568259
A0A024RBU4	hCG_1981838	1.45287	0.00568627
Q53FI7		1.45176	0.00578948
Q59FP4		1.45161	0.00580357
P48735	IDH2	1.45029	0.00592891
B8ZWD9	DBI	1.44804	0.00614846
B4DNW0		1.44755	0.00619727
V9HWG3	HEL-S-45	1.44676	0.00627676
A0A024RBN6	COX6A1	1.44607	0.00634697
A0A0S2Z377	ANXA6	1.44568	0.00638697
P98171	ARHGAP4	1.44432	0.00652836
Q8IV76	PASD1	1.44251	0.0067211
Q9HAJ7	SAP30L	1.44093	0.00689372
P56181	NDUFV3	1.44072	0.00691698
Q92506	HSD17B8	1.44048	0.00694364
O95671	ASMTL	1.44032	0.00696147
A0A024R1Q5	NAGA	1.44005	0.00699166
Q96AQ6	PBXIP1	1.43818	0.00720416
Q9NRZ5	AGPAT4	1.4375	0.00728293
A0A0J9YW36	STMN3	1.43563	0.00750375
Q53HG1		1.43345	0.0077691
P05165	PCCA	1.43303	0.00782122
Q8N7X0	ADGB	1.43267	0.00786617
P29966	MARCKS	1.43034	0.00816295
Q6NS36	FTH1	1.4277	0.00851193
Q9BX40	LSM14B	1.42707	0.00859726
Q96MG8	PCMTD1	1.42659	0.00866281
P37802	TAGLN2	1.42395	0.00903178
Q5U0B9		1.4204	0.00955117
V9HW53	HEL-S-277	1.41951	0.0096857
A0A0S2Z455	SERPINI1	1.41735	0.0100196
Q7Z434	MAVS	1.41657	0.0101428
Q8NDH3	NPEPL1	1.41636	0.0101762
A0A024R5U4	C15orf48	1.41499	0.0103968
Q13308	PTK7	1.41398	0.0105622
Q3T906	GNPTAB	1.41046	0.0111581
Q9UHG3	PCYOX1	1.40984	0.0112663
Q9NZT1	CALML5	1.40919	0.0113808
O95363	FARS2	1.40728	0.0117234
Q15506	SPA17	1.40347	0.0124358
H3BQJ5	NOL3	1.40295	0.012536
O43633	CHMP2A	1.40279	0.0125671
B2R5M8		1.40245	0.0126332
Q9BTT4	MED10	1.40239	0.0126449
B2R4S9	HIST1H2BE	1.40202	0.0127173

(continued on next page)

supplementary table2 (continued)

Uniprot Accession	Gene Symol	Fold Change	P value
A0A024R371	ARL6IP5	1.40161	0.012798
F5GXR3	PTMS	1.39861	0.013403
Q9C0C4	SEMA4C	1.39839	0.0134484
Q0VDC6	FKBP1A	1.39822	0.0134836
A0A087X266	TMEM120A	1.39596	0.0139595
Q9NXJ5	PGPEP1	1.39506	0.0141533
A0A0J9YWL0	AIM1	1.39498	0.0141707
Q7Z4H3	HDDC2	1.39454	0.0142664
V9HWI3	HEL-S-130P	1.39443	0.0142905
Q5HYD7	DKFZp686K101	1.39439	0.0142992
Q9NWM3	CUEDC1	1.39381	0.0144267
B4DSS4		1.39367	0.0144576
Q9BVA1	TUBB2B	1.39334	0.0145307
Q5VU21		1.39323	0.0145552
O95716	RAB3D	1.39301	0.0146042
A0A024RAN2	CAST	1.39078	0.0151097
A0A024RBQ5	OAS3	1.39061	0.0151489
A8K4K1		1.38964	0.0153744
P55085	F2RL1	1.3891	0.0155013
Q9GZV5	WWTR1	1.38497	0.0165042
A0A0S2Z381	ADA	1.38413	0.0167154
B4E2A6		1.38345	0.0168882
P49327	FASN	1.38213	0.0172283
P09382	LGALS1	1.38149	0.0173955
Q32Q14	NDUFA7	1.3809	0.017551
P46939	UTRN	1.38	0.0177906
Q9BVX2	TMEM106C	1.37934	0.0179683
A0A0F7NGI8	LRRFIP1	1.3792	0.0180062
Q13907	IDI1	1.37539	0.0190661
Q9H479	FN3K	1.37472	0.0192584
Q9BTT0	ANP32E	1.37446	0.0193335
E7EV01	CAPN5	1.37384	0.0195136
B2RAU5		1.37375	0.0195399
P49184	DNASE1L1	1.37332	0.0196659
Q8NHP8	PLBD2	1.37303	0.0197513
A0A024QZN7	C10orf70	1.37234	0.0199559
A0A024RA75	HIBADH	1.3722	0.0199976
Q92831	KAT2B	1.37212	0.0200215
Q05639	EEF1A2	1.37204	0.0200454
Q6IAX1	FDFT1	1.37188	0.0200933
P60903	S100A10	1.37058	0.0204865
Q8IY17	PNPLA6	1.36842	0.0211556
B4DVA7		1.36761	0.0214116
Q58WW2	DCAF6	1.36717	0.0215519
V9HWA6	HEL32	1.36682	0.0216641
Q5T749	KPRP	1.3667	0.0217026
Q99943	AGPAT1	1.36638	0.0218058
Q9BPW8	NIPSNAP1	1.36618	0.0218706
B7Z992		1.36461	0.0223849
P20248	CCNA2	1.36421	0.0225177
A0A0K2GN21	BCKDHB	1.36385	0.0226378
P50897	PPT1	1.36294	0.0229442
Q6ZP91		1.36278	0.0229984
A0A0S2Z3Y1	LGALS3BP	1.36156	0.0234159
Q6P1L5	FAM117B	1.36151	0.0234332
D3DTP7	hCG_1987383	1.36075	0.023697
A0A024R0Q4	PLD3	1.36068	0.0237214
Q96CM8	ACSF2	1.36068	0.0237214
A0A024RD07	TNRC5	1.36045	0.0238019
P30044	PRDX5	1.35977	0.0240411
P00167	CYB5A	1.35955	0.0241191
A0A087WVC6	PTPRJ	1.35906	0.0242934

(continued on next page)

supplementary table2 (continued)

Uniprot Accession	Gene Symol	Fold Change	P value
A0A024R943	TOR3A	1.35851	0.0244904
I3L1Y9	FLYWCH2	1.35786	0.0247252
D9YZV5	TPM1	1.35751	0.0248524
Q59GW6		1.35738	0.0248998
B7Z8X5		1.35727	0.02494
Q9BZ23	PANK2	1.35669	0.0251529
Q7Z7A4	PXK	1.35612	0.0253638
L8E8Z4	DST	1.35481	0.0258545
R9S3C3	p14ARF	1.35473	0.0258847
Q86X29	LSR	1.35459	0.0259377
Q59H06		1.35426	0.0260631
Q9UNN8	PROCR	1.3527	0.0266631
H0Y5B0	EPB41L2	1.35234	0.0268034
E7ESZ7	NDUFA10	1.35208	0.0269051
Q5H9L2	TCEAL5	1.35164	0.027078
P62253	UBE2G1	1.35153	0.0271215
P13535	MYH8	1.35129	0.0272163
P11717	IGF2R	1.35011	0.0276873
Q8WVY7	UBLCP1	1.34972	0.0278447
Q9H3H3	C11orf68	1.34881	0.0282149
A0A059QFD5	COX2	1.348	0.0285482
A0A0G2JIW1	HSPA1B	1.34773	0.0286601
Q96NX8	STX16	1.34674	0.0290738
P80217	IFI35	1.34615	0.029323
P60891	PRPS1	1.34551	0.0295954
Q8NCC3	PLA2G15	1.3452	0.0297283
A0A0S2Z3W7	ITPA	1.34372	0.0303698
Q5HYJ3	FAM76B	1.34368	0.0303873
P42773	CDKN2C	1.34345	0.0304882
S6BEP5	HLA-A	1.34308	0.0306511
Q00535	CDK5	1.34305	0.0306643
A0A0A0MT35	FAM213B	1.34298	0.0306953
A0A024R7M1	RFXANK	1.34286	0.0307483
A0A087WY96	SLC6A6	1.34279	0.0307793
A0A024R3H2	SORL1	1.34177	0.0312343
Q30201	HFE	1.34016	0.0319649
Q52LW3	ARHGAP29	1.33876	0.0326128
O95822	MLYCD	1.33865	0.0326642
Q15742	NAB2	1.3385	0.0327344
B9EK46	CGN	1.33806	0.0329412
A0A024R491	C2orf33	1.33801	0.0329648
E7ETM0	CSNK1A1	1.3376	0.0331587
Q5R3I4	TTC38	1.33707	0.0334108
A0A0S2Z522	MYOT	1.33668	0.0335974
Q9BX67	JAM3	1.33663	0.0336215
J3KPT4	TRABD	1.33589	0.0339784
A0A024RDX3	ATP7B	1.33567	0.0340852
O00461	GOLIM4	1.33489	0.0344663
Q6P2D8	XRRA1	1.33366	0.0350751
Q6UXH1	CRELD2	1.33183	0.035999
A0A0C4DFU2	SOD2	1.33152	0.0361577
D3DS14	FLJ10357	1.33062	0.0366218
P26583	HMGB2	1.33024	0.0368195
O15061	SYNM	1.3299	0.0369971
A0A024R5C5	PC	1.32877	0.0375931
Q7Z351	DKFZp686N02209	1.32869	0.0376356
P02649	APOE	1.32846	0.0377581
B2RBV5		1.32821	0.0378916
Q96G28	CFAP36	1.32766	0.038187
Q6IBP4	LAPTM4A	1.32747	0.0382895
P27701	CD82	1.32737	0.0383435
L0R6Q1	SLC35A4	1.32733	0.0383651

(continued on next page)

supplementary table2 (continued)

Uniprot Accession	Gene Symol	Fold Change	P value
P22413	ENPP1	1.32683	0.0386365
Q7L5Y1	ENOSF1	1.32625	0.0389535
P43155	CRAT	1.32545	0.0393946
O14657	TOR1B	1.3253	0.0394778
Q92520	FAM3C	1.32525	0.0395056
Q9NX12		1.32516	0.0395556
Q9H773	DCTPP1	1.32356	0.0404546
P22830	FECH	1.32346	0.0405114
Q9BW83	IFT27	1.32306	0.0407393
A0A024RBL2	SDSL	1.32293	0.0408137
Q9UI15	TAGLN3	1.32151	0.0416334
P12004	PCNA	1.32139	0.0417033
Q9BRA2	TXNDC17	1.32	0.0425213
Q5VV89	MGST3	1.31946	0.042843
Q9BYV8	CEP41	1.31881	0.0432331
A0A1A7UP97	GALNS	1.31877	0.0432572
P42126	ECI1	1.31864	0.0433357
A8K2V5		1.31829	0.0435475
Q9UPY5	SLC7A11	1.31813	0.0436446
Q8IWW6	ARHGAP12	1.31808	0.0436751
M0R189	MPND	1.31797	0.043742
Q15628	TRADD	1.31771	0.0439005
A0A0S2Z433	NDUFS4	1.3177	0.0439067
Q96DH6	MSI2	1.31691	0.0443918
D3DSV6	TEX15	1.31665	0.0445525
P13667	PDIA4	1.31585	0.0450502
Q8TDZ2	MICAL1	1.31545	0.045301
Q14DG0	FBXO18	1.31533	0.0453764
O95425	SVIL	1.31486	0.0456731
Q96Q83	ALKBH3	1.31481	0.0457048
Q9UK58	CCNL1	1.31479	0.0457175
A0A0S2Z5X6	L3MBTL2	1.31469	0.0457809
Q9NZU5	LMCD1	1.31416	0.0461182
Q8N142	ADSSL1	1.31317	0.0467543
B4DJ79		1.31257	0.0471437
A0A024R9V7	LOC92689	1.31257	0.0471437
P49821	NDUFV1	1.31257	0.0471437
Q14679	TTLL4	1.3125	0.0471893
O75879	GATB	1.31031	0.0486364
B7Z6S9		1.31031	0.0486364
P43353	ALDH3B1	1.31027	0.0486632
Q53FA7	TP53I3	1.31007	0.0487973
Q02252	ALDH6A1	1.30988	0.0489251
P0C7P0	CISD3	1.30933	0.0492967
B2R988		1.30863	0.0497733
M0QZ12	GRAMD1A	0.804878	0.0496327
E9PKG0	PLEC	0.804833	0.0495775
A8K6X2		0.803941	0.0484925
A0A024RB85	PA2G4	0.80394	0.0484913
Q96FJ2	DYNLL2	0.8037	0.0482026
Q99426	TBCB	0.803468	0.0479248
Q96G61	NUDT11	0.802548	0.0468357
O00220	TNFRSF10A	0.80248	0.046756
Q68CQ7	GLT8D1	0.802165	0.0463881
Q9UKR5	C14orf1	0.802041	0.046244
B9EH95	ARVCF	0.801786	0.0459486
A8K6U7		0.801164	0.0452343
O15554	KCNN4	0.801053	0.0451078
E7ETZ0	CALM1	0.801029	0.0450805
P17706	PTPN2	0.800922	0.0449588
Q9BSR8	YIPF4	0.800826	0.04485
Q9NRZ7	AGPAT3	0.800805	0.0448261

(continued on next page)

supplementary table2 (continued)

Uniprot Accession	Gene Symol	Fold Change	P value
A0A024R8U7	ET	0.800801	0.0448216
Q96AA3	RFT1	0.800777	0.0447945
Q6FIF5	PXMP4	0.800763	0.0447786
Q5TDH0	DDI2	0.79944	0.0433007
Q96ST8	CEP89	0.79927	0.0431136
Q9H7F0	ATP13A3	0.798677	0.0424662
A8KAH1		0.798442	0.0422118
P47813	EIF1AX	0.79835	0.0421125
A0A0M3R6J9	MAP7D2	0.798222	0.0419747
Q9Y244	POMP	0.798214	0.0419661
Q12792	TWF1	0.798173	0.041922
Q9GZZ9	UBA5	0.798157	0.0419048
A0A024RDS1	HSPH1	0.798025	0.0417633
O95273	CCNDBP1	0.798016	0.0417537
P17812	CTPS1	0.797989	0.0417248
Q8N531	FBXL6	0.797683	0.0413983
G3V161	KBTBD3	0.79702	0.040698
Q14656	TMEM187	0.796926	0.0405995
Q9ULV0	MYO5B	0.79692	0.0405932
A8K2U0	A2ML1	0.796196	0.0398409
P36551	CPOX	0.796086	0.0397276
B4DQ75	DAP	0.795918	0.039555
Q9HCE0	EPG5	0.795475	0.0391028
Q86U90	YRDC	0.795296	0.0389213
Q9UP95	SLC12A4	0.795283	0.0389082
Q8IYH5	ZZZ3	0.795276	0.038901
A0A024R0Q0	FLJ12886	0.79517	0.0387939
A0A024R001	TMEM14C	0.795154	0.0387777
P18077	RPL35A	0.795043	0.0386659
A1A4S6	ARHGAP10	0.794737	0.0383587
E9PR17	CD59	0.794243	0.037867
F8W785	GOLIM4	0.794118	0.0377434
Q96PC3	AP1S3	0.793621	0.0372551
O95210	STBD1	0.79353	0.0371663
Q53HB3		0.793388	0.037028
Q9HD45	TM9SF3	0.792965	0.0366184
Q96HV5	TMEM41A	0.792683	0.0363474
Q8IV53	DENND1C	0.792339	0.036019
Q9NRD1	FBXO6	0.792279	0.035962
K7EL20	EIF3G	0.79174	0.0354528
P15924	DSP	0.791629	0.0353487
Q5ZEY3	GAPD	0.791513	0.0352402
Q9GZN1	ACTR6	0.789937	0.0337919
B4DR61	SEC61A1	0.789907	0.0337648
A0A087X1K4	OR3A1	0.789623	0.0335092
O60488	ACSL4	0.789568	0.0334598
F8W7C6	RPL10	0.789474	0.0333757
Q14376	GALE	0.789315	0.0332337
F8WES2	MTAP	0.789062	0.0330088
P51571	SSR4	0.78876	0.032742
Q9NZV1	CRIM1	0.788328	0.0323633
P50402	EMD	0.787453	0.0316071
Q6PI26	SHQ1	0.786997	0.0312188
Q96LD1	SGCZ	0.786089	0.030457
D6RB85	CANX	0.785777	0.0301988
A0A0A6YY96	IREB2	0.785714	0.0301469
A8K9K8		0.784656	0.0292856
A0A0C4DFM2	ZNF574	0.784404	0.0290834
A0A024R0W3	SLC38A2	0.784181	0.0289055
Q6ZSJ8	C1orf122	0.783394	0.0282846
P49770	EIF2B2	0.782847	0.0278595
B1AKK2	DDAH1	0.782691	0.0277392

(continued on next page)

supplementary table2 (continued)

Uniprot Accession	Gene Symol	Fold Change	P value
Q8ND82	ZNF280C	0.782486	0.0275818
Q8N806	UBR7	0.782412	0.0275251
P24311	COX7B	0.782027	0.027232
Q9BT40	INPP5K	0.781219	0.026625
Q9UNL2	SSR3	0.780888	0.0263796
Q9NP72	RAB18	0.780797	0.0263124
O00267	SUPT5H	0.780622	0.0261837
Q13501	SQSTM1	0.780396	0.0260182
Q59HH7		0.780369	0.0259984
A0A024RBA9	RAB21	0.779453	0.0253368
A0A024R0N7	SAMD4B	0.779233	0.02518
A0A0A1HAV6		0.778872	0.0249244
Q01469	FABP5	0.778819	0.024887
P46976	GYG1	0.778704	0.0248061
Q8IY57	YAF2	0.777885	0.0242363
F5H136	TCP1	0.777403	0.023906
Q9UBQ5	EIF3K	0.777385	0.0238938
P62834	RAP1A	0.777219	0.0237809
Q587J7	TDRD12	0.777188	0.0237599
B3KNS4	ERVK3-1	0.777003	0.0236348
Q92685	ALG3	0.776482	0.0232852
Q59ET0		0.776471	0.0232779
A0A024R2W4	DAG1	0.776316	0.0231748
Q9NR30	DDX21	0.776268	0.0231429
Q8WUI4	HDAC7	0.776177	0.0230826
O95059	RPP14	0.775878	0.0228854
P26038	MSN	0.775746	0.0227987
A0A024R7K6	FKSG24	0.774536	0.0220171
Q5T094	RER1	0.774257	0.02184
Q01968	OCRL	0.774194	0.0218002
Q9UBV7	B4GALT7	0.774135	0.021763
E9LUH4	MECP2	0.774034	0.0216994
A8K1U0		0.773356	0.0212763
P02795	MT2A	0.77305	0.0210876
B2RCP7		0.77193	0.0204086
Q99808	SLC29A1	0.771632	0.0202311
Q8TCF1	ZFAND1	0.770872	0.019784
Q16533	SNAPC1	0.770563	0.0196046
A0A0A6YYH1	C15orf38-AP3S2	0.770042	0.0193052
O95707	POP4	0.769713	0.019118
A0A024RAG1	MGC4268	0.769643	0.0190784
B4DR48		0.76938	0.0189302
B8ZZD4	TAX1BP1	0.769231	0.0188466
P53611	RABGGTB	0.769097	0.0187717
P17096	HMGA1	0.767928	0.0181288
Q8N9Q2	SREK1IP1	0.76781	0.018065
A0A024R355	THEX1	0.76779	0.0180542
H7C1Q3	HHATL	0.766816	0.0175344
Q70JA7	CHSY3	0.766667	0.017456
Q8WUP2	FBLIM1	0.766402	0.0173173
Q99986	VRK1	0.766397	0.0173147
A0A024RA87	7-Sep	0.765858	0.0170354
A0A096LP99	PRAMEF18	0.765405	0.0168036
B9EGE7	ZNF507	0.764286	0.0162423
Q2PPJ7	RALGAPA2	0.763265	0.0157439
E7EQ64	PRSS1	0.763069	0.0156497
P35247	SFTPD	0.762869	0.015554
A0A1B0GU57	TDRD12	0.762288	0.015279
Q659C4	LARP1B	0.761578	0.0149484
Q8N9M1	C19orf47	0.761152	0.014753
P05198	EIF2S1	0.761086	0.0147229
Q86TV6	TTC7B	0.760956	0.0146638

(continued on next page)

supplementary table2 (continued)

Uniprot Accession	Gene Symol	Fold Change	P value
Q5H924	HUWE1	0.760835	0.0146089
Q96C19	EFHD2	0.760692	0.0145443
Q96EF6	FBXO17	0.759207	0.0138876
O15446	CD3EAP	0.758748	0.0136897
Q9UJ68	MSRA	0.758685	0.0136628
Q9UL15	BAG5	0.758467	0.0135698
Q12894	IFRD2	0.7584	0.0135413
Q9HD64	XAGE1A	0.758065	0.0133997
G3V1M7	ACADVL	0.757812	0.0132936
O43665	RGS10	0.756976	0.012948
B6CAV5	KIR2DS5	0.756661	0.0128197
A0A024R601	CLN6	0.756139	0.0126095
A0A1B0GUD6	GRAMD1B	0.75605	0.012574
A0A0D9SGE8	PHF6	0.755703	0.0124362
Q9NS00	C1GALT1	0.755535	0.01237
A0A0A0MTS2	GPI	0.755319	0.0122853
A0A024R9N6	EHD4	0.754734	0.0120582
Q9P2S5	WRAP73	0.754697	0.012044
G3V153	CAPRIN1	0.754113	0.0118211
Q05513	PRKCZ	0.753932	0.0117528
Q59HE8		0.753927	0.0117509
A0A024RAE1	C1orf33	0.753888	0.0117362
O94827	PLEKHG5	0.752587	0.0112552
B7ZMD6	IRGQ	0.752051	0.0110619
P46734	MAP2K3	0.751958	0.0110287
P29317	EPHA2	0.750951	0.0106741
A0A024RAM4	MAP1B	0.750457	0.0105037
A8K9T5		0.749767	0.0102696
Q9H2K2	TNKS2	0.749603	0.0102146
B7ZKQ8	PODXL	0.748187	0.00975016
J3KPZ4	C1D	0.748099	0.0097219
Q14627	IL13RA2	0.747938	0.00967036
Q5T6V5	C9orf64	0.747715	0.00959939
A0A024R8G6	SSNA1	0.745931	0.00904732
P00492	HPRT1	0.745748	0.00899224
Q9UPI5	PLAUR	0.74547	0.00890914
O95433	AHSA1	0.745081	0.00879395
A0A024R1S8	LASP1	0.744098	0.00850852
Q14061	COX17	0.744094	0.00850737
O96033	MOCS2	0.74359	0.00836413
Q03405	PLAUR	0.743491	0.00833624
A0A087WT10	APITD1-CORT	0.743339	0.00829357
Q8NI62	OK/KNS-cl.6	0.742906	0.00817303
P36954	POLR2I	0.742555	0.00807645
P41208	CETN2	0.741907	0.00790066
P51617	IRAK1	0.741563	0.00780869
A0A0C4DG49	PVR	0.74143	0.00777337
Q15434	RBMS2	0.740707	0.00758378
Q16222	UAP1	0.739703	0.0073271
Q9Y6I4	USP3	0.738854	0.00711588
Q9BUF5	TUBB6	0.738408	0.00700705
Q8IWC1	MAP7D3	0.738117	0.0069368
Q9BRP1	PDCD2L	0.737676	0.00683151
B4E0Y9	STK26	0.73766	0.0068277
Q8N7G1		0.736986	0.0066695
A4D1F3	LOC392745	0.735786	0.00639564
B2RWP0	SIPA1L3	0.734226	0.00605413
Q59ED5		0.733962	0.00599792
H2DF05	IL10RA	0.732611	0.00571726
A8K5C2		0.732472	0.00568904
A0A024R0Q5	PPP1R13L	0.732435	0.00568154
A0A024R658	ZFP36L1	0.731641	0.00552281

(continued on next page)

supplementary table2 (continued)

Uniprot Accession	Gene Symol	Fold Change	P value
Q96DW6	SLC25A38	0.731383	0.00547207
P12429	ANXA3	0.731357	0.00546697
P26022	PTX3	0.731118	0.00542037
Q3KQV9	UAP1L1	0.72973	0.0051564
Q96CX6	LRRC58	0.72908	0.00503666
P61225	RAP2B	0.72686	0.00464568
P55010	EIF5	0.725	0.00433866
Q96GX2	ATXN7L3B	0.723461	0.0040981
P43356	MAGEA2	0.721448	0.00380105
Q9H6K4	OPA3	0.721182	0.00376323
Q9BVK8	TMEM147	0.721063	0.00374643
P21333	FLNA	0.720602	0.00368192
Q9Y5Z9	UBIAD1	0.719298	0.00350468
Q86WQ0	NR2C2AP	0.718808	0.00344002
A0A024QZF8	EMP3	0.718657	0.00342031
A0A024R5J6	CHCHD8	0.718057	0.00334296
Q5VUJ6	LRCH2	0.718023	0.00333862
B2RBX9		0.716592	0.00316049
A8MT69	STRA13	0.715799	0.00306539
Q86SE9	PCGF5	0.71544	0.00302317
P11441	UBL4A	0.714549	0.00292059
K7EJ96	ROGDI	0.714475	0.00291221
A8K556		0.714418	0.00290577
Q0D2I6	FEZ2	0.713928	0.00285091
Q8IXW5	RPAP2	0.713918	0.0028498
Q9UQ03	CORO2B	0.71193	0.0026365
A0A024R0P9	TOMM40	0.711255	0.00256735
C4P096	DISC1	0.710692	0.00251088
P19623	SRM	0.709562	0.00240084
Q9H098	FAM107B	0.708729	0.00232247
Q16831	UPP1	0.707826	0.00224006
P05412	JUN	0.707452	0.00220669
A0A024R0L5	GSK3A	0.707361	0.00219863
Q9P2N7	KLHL13	0.707062	0.00217235
P04731	MT1A	0.704803	0.00198261
O43707	ACTN4	0.70461	0.0019671
P60468	SEC61B	0.703256	0.00186126
B2R6I6		0.701395	0.00172401
Q86TX8		0.701266	0.00171484
B9EIK3	DDX26B	0.699057	0.00156433
Q9BWH2	FUNDC2	0.697987	0.00149573
O96005	CLPTM1	0.697987	0.00149573
P30530	AXL	0.697077	0.0014395
Q9NSY1	BMP2K	0.69678	0.00142156
Q9GZS1	POLR1E	0.695922	0.00137084
A0A1B0GV05	RAP1GAP2	0.695614	0.00135303
D3DWX8	FAM3A	0.695489	0.00134586
O75145	PPFIA3	0.694859	0.00131024
P04083	ANXA1	0.693192	0.00121999
B4DLE6		0.690759	0.0010982
Q9Y3L5	RAP2C	0.690499	0.00108585
Q8IYD8	FANCM	0.687914	0.000969617
Q96D05	C10orf35	0.687791	0.000964373
Q9H8P0	SRD5A3	0.685393	0.000867099
P78358	CTAG1A	0.684811	0.000844857
Q8IXJ6	SIRT2	0.682511	0.000761865
H0YG38	PRPF40A	0.68231	0.000754973
A0A087WVP4	SMTN	0.682196	0.000751089
A0A024R5Z9	PKM2	0.681287	0.000720747
Q96DU7	ITPKC	0.680352	0.00069069
P27469	G0S2	0.67801	0.00062028
A0A024RAM2	GLRX	0.677515	0.000606251

(continued on next page)

supplementary table2 (continued)

Uniprot Accession	Gene Symol	Fold Change	P value
Q8N4H5	TOMM5	0.677388	0.000602699
Q9BW60	ELOVL1	0.676316	0.000573445
Q14315	FLNC	0.675893	0.000562257
Q9BVJ7	DUSP23	0.675676	0.000556594
Q4G148	GXYLT1	0.675439	0.000550469
A4IF29	UST6	0.674528	0.000527483
A8K8D9		0.67288	0.000488086
Q8IUS5	EPHX4	0.672163	0.000471789
A2VCK8	TMSB4X	0.670068	0.000426944
G8I2S8		0.668449	0.000394963
Q8N2A8	PLD6	0.668333	0.000392757
P31150	GDI1	0.666972	0.000367692
Q99584	S100A13	0.666667	0.000362278
B2R9B4		0.665906	0.000349081
Q9H8M7	FAM188A	0.661319	0.000278336
P51572	BCAP31	0.659981	0.000260304
P55809	OXCT1	0.658017	0.000235751
Q8NE91	TM4SF1	0.652535	0.000177935
Q5VT66	1-Mar	0.649208	0.000149477
O00151	PDLIM1	0.649074	0.000148423
A0A024QYT5	SERPINE1	0.645744	0.000124314
Q15738	NSDHL	0.645075	0.000119926
A0A024RC10	hCG_1740677	0.64168	9.98E-05
J3KQN4	RPL36A	0.637655	7.99E-05
O14737	PDCD5	0.636598	7.53E-05
A0A075B6G3	DMD	0.633248	6.24E-05
J3KSZ0	EIF4A1	0.627586	4.50E-05
A0A087WXL3	POLQ	0.626536	4.23E-05
B4DW33		0.62605	4.11E-05
A0A0C4DFN3	MGLL	0.620893	3.03E-05
Q9NP84	TNFRSF12A	0.619125	2.72E-05
Q8N6I1	EID2	0.618907	2.69E-05
Q14320	FAM50A	0.617306	2.44E-05
A0A140VJM4	KCTD12	0.616667	2.34E-05
O60344	ECE2	0.615442	2.17E-05
B9VJ61	TLR5	0.609558	1.50E-05
Q86VH2	KIF27	0.606205	1.21E-05
Q9NSC2	SALL1	0.604594	1.09E-05
Q96FQ6	S100A16	0.603545	1.02E-05
I3L3X0	ZG16B	0.600285	8.25E-06
F8VX04	SLC38A1	0.598738	7.45E-06
O43719	HTATSF1	0.598598	7.38E-06
P43360	MAGEA6	0.597122	6.68E-06
A0A087WUC3	SCNN1D	0.589944	4.10E-06
A0A024QZM9	PLAU	0.580645	2.12E-06
O00373		0.577815	1.73E-06
Q9P0U1	TOMM7	0.573786	1.28E-06
A0A024RDD9	CXCL2	0.571645	1.09E-06
P0C5Z0	H2AFB2	0.564126	6.13E-07
Q7RTU9	STRC	0.544828	1.27E-07
P24390	KDELR1	0.542857	1.07E-07
Q8TBS0	C18orf21	0.539749	8.18E-08
O00622	CYR61	0.537358	6.63E-08
Q60FE5	FLNA	0.528787	3.07E-08
P10620	MGST1	0.52462	2.08E-08
O95567	C22orf31	0.514901	8.21E-09
P56277	CMC4	0.5109	5.53E-09
B2R9M7		0.498512	1.55E-09

supplementary table3

Differential expressed proteins by iTARQ coupled LC-MS/MS analysis from H1299-shUSP35-2 vs.H1299-scramble

Uniprot Accession	Gene Symol	Fold Change	P value
G3V1M7	ACADVL	8.19585	5.93E-194
O00373		4.27163	4.78E-94
Q7RTU9	STRC	4.02265	1.16E-86
P05161	ISG15	3.40596	8.53E-68
D9ZGG2	VTN	3.26235	2.69E-63
P09914	IFIT1	2.72489	3.35E-46
I3L3X0	ZG16B	2.69063	4.20E-45
Q6P2D8	XRRA1	2.43041	9.02E-37
Q8NE91	TM4SF1	2.42702	1.16E-36
B2R9M7		2.41781	2.28E-36
O95567	C22orf31	2.36423	1.16E-34
Q5T765	IFIT3	2.24935	5.11E-31
Q5VT66	1-Mar	2.2399	1.02E-30
K7EJ96	ROGDI	2.2009	1.72E-29
Q9HB65	ELL3	2.18215	6.67E-29
B3VL31		2.17609	1.03E-28
C4P096	DISC1	2.11806	6.72E-27
A0A087WUC3	SCNN1D	2.06786	2.42E-25
Q9GZN1	ACTR6	2.01051	1.41E-23
H0Y7R8	AFDN	2.00895	1.57E-23
P62745	RHOB	1.81807	8.16E-18
Q0D2M7	DNAJC13	1.81062	1.35E-17
Q9NXG6	P4HTM	1.80694	1.72E-17
Q8IZQ1	WDFY3	1.80425	2.06E-17
A0A024QYX0	EBP	1.78875	5.79E-17
Q8IY21	DDX60	1.78069	9.89E-17
H7C114	PCLO	1.76196	3.41E-16
Q6AZW6	ATF6B	1.72055	5.10E-15
Q53HB4		1.71267	8.49E-15
A0A0A0MRJ7	F5	1.70568	1.33E-14
Q86VH2	KIF27	1.69543	2.57E-14
A4IF29	UST6	1.65808	2.75E-13
O95837	GNA14	1.65097	4.30E-13
P24390	KDELR1	1.61792	3.35E-12
P15531	NME1	1.59172	1.66E-11
P09466	PAEP	1.58968	1.88E-11
O95786	DDX58	1.58353	2.72E-11
P58004	SESN2	1.56617	7.70E-11
Q59GW5		1.56083	1.06E-10
A0A0F7G8J1	PLG	1.55986	1.12E-10
A8K304		1.53102	6.09E-10
B2R7U4		1.52784	7.32E-10
Q9UE89		1.52745	7.49E-10
A0A024QZU8	RREB1	1.52547	8.40E-10
A0A024R3E3	APOA1	1.52022	1.14E-09
Q8NEN9	PDZD8	1.51754	1.33E-09
Q495P6	TLR8	1.51106	1.92E-09
A6NCK2	TRIM43B	1.50835	2.24E-09
A0A0B6XJY0	CASK	1.50779	2.32E-09
A5Z217		1.5059	2.58E-09
A5A3E0	POTEF	1.50236	3.16E-09
Q13488	TCIRG1	1.50175	3.27E-09
Q9NWA0	MED9	1.50136	3.34E-09
Q5CZB5	DKFZp686M0430	1.48235	9.74E-09
Q9P2H3	IFT80	1.47686	1.32E-08
Q7Z351	DKFZp686N02209	1.47091	1.84E-08
Q96MH2	HEXIM2	1.47052	1.88E-08
Q9UII4	HERC5	1.46732	2.24E-08
B5MEA4	ANKRD12	1.4578	3.77E-08
Q9H9J5		1.45703	3.94E-08
Q969T4	UBE2E3	1.45595	4.17E-08
P02533	KRT14	1.4547	4.47E-08

(continued on next page)

supplementary table3 (continued)

Uniprot Accession	Gene Symol	Fold Change	P value
A0A059RHF6	COX1	1.44236	8.71E-08
Q5BIX2	ARKL1	1.43905	1.04E-07
Q8NC51	SERBP1	1.43546	1.26E-07
P62380	TBPL1	1.43417	1.35E-07
A0A024R001	TMEM14C	1.43077	1.62E-07
H9S5Y2	COX2	1.42821	1.85E-07
B4DR60		1.42687	1.99E-07
P05412	JUN	1.42534	2.16E-07
P02786	TFRC	1.41818	3.14E-07
B7Z4T6		1.41337	4.04E-07
Q8TCT8	SPPL2A	1.40909	5.04E-07
B4DZK2		1.40892	5.09E-07
Q59ET0		1.40496	6.24E-07
Q96K80	ZC3H10	1.40481	6.29E-07
P15559	NQO1	1.4021	7.24E-07
B4DM67		1.39728	9.26E-07
B3KN09		1.39588	9.95E-07
G3V161	KBTBD3	1.3903	1.32E-06
Q5HYJ3	FAM76B	1.38855	1.44E-06
A0A024RBV9	TBL1X	1.38293	1.92E-06
H0YBL0	CDH17	1.3826	1.95E-06
I7H1S4	COX3	1.38209	2.00E-06
Q8NFU1	BEST2	1.37701	2.58E-06
Q8N2U0	TMEM256	1.37634	2.67E-06
O14521	SDHD	1.37204	3.30E-06
Q9NQR7	CCDC177	1.37051	3.56E-06
P13533	MYH6	1.36986	3.68E-06
Q8N7X0	ADGB	1.36605	4.44E-06
B5BU36	TNFRSF10B	1.36153	5.54E-06
P13647	KRT5	1.35929	6.18E-06
B2RBH6		1.359	6.26E-06
Q9BTV5	FSD1	1.3586	6.39E-06
A0A024RB05	CD63	1.35368	8.11E-06
Q3KQV9	UAP1L1	1.35239	8.63E-06
A0PJW6	TMEM223	1.35211	8.75E-06
Q9ULS5	TMCC3	1.3512	9.14E-06
H7BY16	NCL	1.35034	9.53E-06
Q8N2H4	SYS1	1.35023	9.58E-06
D6RIT2	HNRNPH1	1.34783	1.08E-05
A0A161I202	LTF	1.3341	2.06E-05
Q9BQT8	SLC25A21	1.33333	2.14E-05
A0A024RDJ1	DC2	1.32908	2.61E-05
B3KSH8		1.32772	2.78E-05
A8MYA2	CXorf49	1.32235	3.57E-05
P48507	GCLM	1.31567	4.85E-05
G3CIG1	MUC19	1.31284	5.51E-05
B3KSB5		1.30686	7.22E-05
B4E0X8		1.3051	7.82E-05
Q96NB2	SFXN2	1.30435	8.09E-05
Q96A26	FAM162A	1.30328	8.48E-05
P0CG38	POTEI	1.30307	8.57E-05
Q96BI3	APH1A	1.29814	0.000106652
Q9P0U1	TOMM7	1.29758	0.000109329
P28562	DUSP1	1.29589	0.000117852
P15336	ATF2	1.29581	0.00011828
H9NKY4	IFITM3	1.29205	0.000139523
C0JYY2	APOB	1.29082	0.000147299
B4DVS4		1.29077	0.000147591
H0Y9L1	NEDD4	1.28878	0.000161032
Q562M3	ACT	1.28874	0.000161278
Q8IUH5	ZDHHC17	1.28636	0.000178906
I7GW38	ND3	1.28619	0.000180207

(continued on next page)

supplementary table3 (continued)

Uniprot Accession	Gene Symol	Fold Change	P value
B4E1J2		1.28523	0.000187875
P13645	KRT10	1.2785	0.000251111
Q5TG30	ARHGAP40	1.27832	0.00025304
A0A024R911	XPR1	1.27618	0.000277226
B4DR61	SEC61A1	1.27565	0.000283503
Q9BVK8	TMEM147	1.27235	0.000326192
A8K6Q8		1.2722	0.000328309
F5H265	UBC	1.26835	0.00038614
A0A059QQM1	ATP6	1.26745	0.000400855
R4GMX5	BSG	1.26419	0.000459422
I6L8A6	RBBP8	1.2618	0.000507315
B2RBJ7		1.26157	0.000512193
A8KAH1		1.26155	0.000512553
Q9Y644	RFNG	1.26132	0.000517469
Q9BZE2	PUS3	1.26015	0.000543128
A0A024QZF8	EMP3	1.259	0.000569602
Q8WXV6	PLEC1	1.25878	0.000574874
O95359	TACC2	1.25829	0.000586563
Q6P2P2	PRMT9	1.2581	0.000591088
B4DRA2		1.25799	0.000593759
E9PL71	EEF1D	1.25524	0.000664641
Q7Z7E8	UBE2Q1	1.25461	0.000681913
A8K4T9		1.25264	0.000738906
A0A087WVA1	SELT	1.25025	0.000814109
Q99541	PLIN2	1.24893	0.000858766
O15431	SLC31A1	1.24804	0.000890339
A4D1W6	C7orf11	1.24626	0.000956213
B2RDY3		1.24608	0.00096316
A0A024R0W3	SLC38A2	1.24583	0.000973014
A0A024RDX3	ATP7B	1.24552	0.000985173
B4E388		1.2398	0.00123674
Q9Y3D5	MRPS18C	1.23481	0.0015049
B2RWP4	TACC2	1.23427	0.00153745
Q53GL6	RALY	1.23341	0.00158968
F8WB05	ATXN2	1.23303	0.00161327
Q59EW6		1.23212	0.00167125
D6W601	ADAT3	1.23123	0.00173023
Q9NS00	C1GALT1	1.23085	0.00175592
Q5K651	SAMD9	1.23037	0.00178882
A0A024R8T9	SYNGR2	1.23006	0.00181046
Q71DI3	HIST2H3A	1.2296	0.00184277
P48436	SOX9	1.22825	0.00194161
F8VTV8	CDK4	1.22779	0.00197626
K7EJB5	SNRPD2	1.22727	0.002016
Q13501	SQSTM1	1.22679	0.00205348
O43422	THAP12	1.22662	0.00206697
Q8NEA6	GLIS3	1.22584	0.00212961
H0YMV8	RPS27L	1.22514	0.00218755
Q02952	AKAP12	1.22357	0.00232306
A4D126	ISPD	1.22326	0.00235032
Q8IYD8	FANCM	1.22222	0.00244512
P15882	CHN1	1.22208	0.00245872
Q9NSC2	SALL1	1.22174	0.00249036
Q96F46	IL17RA	1.22135	0.00252723
Q9BVN2	RUSC1	1.22103	0.00255821
Q6P161	MRPL54	1.22092	0.00256923
A0A0X7YVE4	HLA-C	1.22019	0.00264035
H6VRG1	KRT1	1.21908	0.00275421
Q6ZP91		1.21899	0.00276343
P29401	TKT	1.21725	0.00295052
Q16533	SNAPC1	1.21655	0.00302806
D3DS77	KIAA0323	1.21625	0.00306282

(continued on next page)

supplementary table3 (continued)

Uniprot Accession	Gene Symol	Fold Change	P value
A0A1B0GUH9	LCOR	1.2156	0.00313786
P42677	RPS27	1.21509	0.00319835
A0A024R5J6	CHCHD8	1.21455	0.00326315
A8K725		1.21366	0.00337279
H7C1Q3	HHATL	1.21242	0.00353255
H0YDV2	CCDC73	1.21167	0.00363176
Q2TSD0		1.21147	0.00365886
Q9NRZ5	AGPAT4	1.21143	0.00366358
Q9P2C4	TMEM181	1.21113	0.00370447
Q9BVA1	TUBB2B	1.21066	0.00376951
B4DSI9		1.21047	0.0037968
Q6QNY1	BLOC1S2	1.20892	0.00401873
Q14061	COX17	1.20594	0.00448262
H0Y4U4	PAEP	1.20532	0.00458528
Q96AB3	ISOC2	1.20513	0.00461726
Q9BRS2	RIOK1	1.20452	0.00472055
Q9H582	ZNF644	1.20368	0.00486577
Q9H0H3	KLHL25	1.20348	0.00490167
H0Y5B0	EPB41L2	1.20339	0.00491806
B7ZVY3	GAGE4	1.20294	0.00499803
Q6P4Q7	CNNM4	1.2	0.00555754
Q0P5W4	RCE1	1.2	0.00555754
Q0PNE2	ELP6	0.833333	0.0449174
B3KWQ9		0.833192	0.0447005
Q9UHP3	USP25	0.833034	0.0444584
O75319	DUSP11	0.832889	0.044237
Q8IY22	CMIP	0.832856	0.0441866
Q16626	MEA1	0.83285	0.0441781
Q8TEQ8	PIGO	0.832734	0.0440015
B4DNA3		0.832669	0.0439039
P31273	HOXC8	0.832517	0.0436742
Q13946	PDE7A	0.832432	0.0435468
Q53GT1	KLHL22	0.832161	0.0431403
Q15468	STIL	0.832124	0.043086
Q9BQD7	FAM173A	0.831599	0.042309
A0A024R8I8	C9orf140	0.831291	0.0418595
Q6PI26	SHQ1	0.83046	0.0406638
I3NI25	FOPNL	0.830009	0.0400269
P33981	TTK	0.829767	0.0396883
B4E2F2		0.829767	0.0396896
P41229	KDM5C	0.829746	0.0396591
P04350	TUBB4A	0.829736	0.0396461
A0A087WT10	APITD1-CORT	0.829443	0.0392402
Q15800	MSMO1	0.828571	0.0380514
Q9UHD8	9-Sep	0.828496	0.03795
D6W4Z6	hCG_23833	0.828452	0.0378907
B7Z1N4		0.828383	0.0377982
Q9NYG5	ANAPC11	0.828358	0.0377652
A8K322		0.828322	0.0377168
Q9NQ11	ATP13A2	0.828167	0.0375095
Q53EQ6	TIGD5	0.828014	0.0373072
P17483	HOXB4	0.827907	0.0371654
A1A4S6	ARHGAP10	0.827877	0.0371252
B3KWY9		0.827751	0.0369602
H7BYJ1	RNF34	0.827681	0.0368679
Q9NWW7	C2orf42	0.827586	0.0367438
A0A0S2Z455	SERPINI1	0.827521	0.0366588
A8K383		0.827381	0.036476
Q9NZN1	IL1RAPL1	0.827139	0.0361626
H8Y6P7	GCOM1	0.826691	0.0355882
E7EQB3	TSEN34	0.82668	0.0355732
Q96QE3	ATAD5	0.826561	0.0354223

(continued on next page)

supplementary table3 (continued)

Uniprot Accession	Gene Symol	Fold Change	P value
Q9UBV7	B4GALT7	0.826512	0.0353607
A8K313		0.826511	0.0353585
Q9Y448	KNSTRN	0.826389	0.0352043
O95551	TDP2	0.826206	0.0349734
A0A024R0Q6	ERCC1	0.826167	0.0349243
A0A140LJL2	CBARP	0.82579	0.0344543
P43353	ALDH3B1	0.825411	0.033987
D6W5U7	STAG3	0.824832	0.0332815
F8W0Q9	PPHLN1	0.82453	0.0329194
Q9BSR8	YIPF4	0.824434	0.0328044
Q92896	GLG1	0.823899	0.0321725
Q96H35	RBM18	0.823851	0.032116
Q8WXH0	SYNE2	0.823684	0.0319209
Q96E14	RMI2	0.823579	0.0317988
O75147	OBSL1	0.823529	0.0317409
Q8IY45	AMN1	0.823256	0.0314248
B2RNT7	KLHDC5	0.823049	0.0311875
D3DS14	FLJ10357	0.822993	0.0311232
B2RD96		0.822938	0.0310604
H7BYT1	CSNK1D	0.822857	0.0309688
P54753	EPHB3	0.822796	0.0308994
O43909	EXTL3	0.822763	0.0308616
A0A0S2Z4N0	MEF2A	0.821866	0.0298589
P54821	PRRX1	0.821818	0.029806
P15924	DSP	0.821525	0.0294842
A0A024R6G6	NDUFB1	0.821313	0.0292542
B2RC06		0.821285	0.0292237
A0A0A0MTL5	SKP2	0.821083	0.0290055
Q15796	SMAD2	0.821053	0.0289726
Q58A45	PAN3	0.820882	0.0287896
Q9P021	CRIPT	0.820489	0.0283711
Q5TF21	SOGA3	0.820276	0.0281474
O14777	NDC80	0.82	0.0278582
Q6PIJ6	FBXO38	0.819168	0.0270029
Q2PPJ7	RALGAPA2	0.819055	0.0268886
Q96AP0	ACD	0.818949	0.0267816
Q9NYZ3	GTSE1	0.818905	0.0267374
D3DR40	C10orf4	0.818613	0.0264448
A0A024R7K9	SSBP4	0.818584	0.0264155
Q17RV3	LRRK2	0.818522	0.0263537
Q9Y654	CBX1	0.818489	0.0263212
X6REH9	UBE2W	0.818475	0.0263068
Q9UHB7	AFF4	0.818426	0.0262582
P55085	F2RL1	0.817869	0.0257113
Q9H000	MKRN2	0.817857	0.0256993
H3BND9	PCBP2	0.817793	0.0256369
B3KW05		0.817621	0.0254703
H0Y9P0	RACK1	0.817121	0.0249902
Q9NWM3	CUEDC1	0.817013	0.0248879
A7LNJ1	SLC20A1	0.816189	0.0241167
A8K2A1	hCG_2030297	0.815812	0.0237709
A0A023T6R1	FLJ10292	0.815526	0.0235107
Q9UMX1	SUFU	0.815081	0.023111
Q562Z4	ACT	0.814558	0.022649
O75787	ATP6AP2	0.813953	0.0221243
P33897	ABCD1	0.813389	0.0216437
Q5ZEY3	GAPD	0.813216	0.0214979
Q9UBN6	TNFRSF10D	0.813075	0.0213804
B3KPL2	PHF15	0.812727	0.0210913
Q13393	PLD1	0.812444	0.0208588
Q96CG8	CTHRC1	0.812399	0.020822
Q15417	CNN3	0.812383	0.0208084

(continued on next page)

supplementary table3 (continued)

Uniprot Accession	Gene Symol	Fold Change	P value
E5RJF8	CETN3	0.811975	0.0204776
Q86YQ8	CPNE8	0.811881	0.0204024
Q9UKK3	PARP4	0.811556	0.0201423
Q12834	CDC20	0.811224	0.0198808
J3KP02	LEKR1	0.811047	0.0197414
H3BTB6	CMC2	0.811038	0.019735
Q53FS1		0.810614	0.019406
P84022	SMAD3	0.810362	0.0192126
Q8WUP2	FBLIM1	0.810261	0.0191356
M0QY77	KXD1	0.810212	0.0190986
Q9H3U5	MFSD1	0.809991	0.0189315
Q7Z406	MYH14	0.809361	0.0184613
A0A0A0MR57	ZNF836	0.809339	0.0184449
A0A0A0MQR2	RTFDC1	0.808955	0.0181641
Q9H3F6	KCTD10	0.808728	0.0179992
Q14680	MELK	0.808654	0.0179458
J3KN59	BNIP2	0.808247	0.0176549
O14757	CHEK1	0.808247	0.0176549
P04066	FUCA1	0.808134	0.0175747
Q15506	SPA17	0.807903	0.0174119
B3KX14		0.807729	0.01729
Q8IZT6	ASPM	0.807551	0.0171656
Q9BVS5	TRMT61B	0.807229	0.0169435
Q06609	RAD51	0.806773	0.0166329
Q9H0I2	ENKD1	0.806061	0.0161575
O15240	VGF	0.806011	0.0161248
B6ZGT0	NR1I1	0.805583	0.0158454
Q66GS9	CEP135	0.805479	0.0157782
X6REB3	TCF3	0.805147	0.0155646
B2RBX9		0.804498	0.0151549
P46937	YAP1	0.804444	0.0151213
Q96F85	CNRIP1	0.804367	0.015073
Q7Z5U6	WDR53	0.80431	0.0150379
E5KTI5	NTHL1	0.804143	0.0149346
Q5T0D9	TPRG1L	0.804143	0.0149346
Q07820	MCL1	0.80387	0.0147668
O00506	STK25	0.803648	0.0146319
Q9Y248	GINS2	0.803554	0.0145749
B3KXW2		0.803381	0.0144707
Q8WUW1	BRK1	0.80315	0.0143325
Q08999	RBL2	0.803109	0.0143082
A0A0B4J1V9	HELLS	0.803059	0.0142788
Q96CX6	LRRC58	0.803016	0.0142531
O75794	CDC123	0.803015	0.0142526
A8K6X2		0.802993	0.0142392
A0A140VJU4		0.802643	0.0140336
Q9Y275	TNFSF13B	0.80241	0.0138976
D3DSQ0	PCM1	0.801668	0.0134733
Q63ZY3	KANK2	0.801043	0.0131238
A0A024R254	MAGED1	0.800725	0.0129492
B4DRE5		0.800551	0.012855
B0QYU2	CXorf39	0.800238	0.0126857
A0A024R0B3	C1orf86	0.800189	0.0126598
Q14681	KCTD2	0.800182	0.0126561
Q9ULV0	MYO5B	0.8	0.0125587
P49638	TTPA	0.799807	0.0124562
Q9NVP2	ASF1B	0.799801	0.0124531
A1A5C5	RRBP1	0.799122	0.012099
O14965	AURKA	0.798483	0.0117738
Q96GD4	AURKB	0.797798	0.0114331
E9PDU6	CNN3	0.797657	0.0113643
Q53H80	AKIRIN2	0.79686	0.0109806

(continued on next page)

supplementary table3 (continued)

Uniprot Accession	Gene Symol	Fold Change	P value
A0A0S2Z542	MAPK13	0.796756	0.0109315
O60437	PPL	0.796233	0.010687
Q16763	UBE2S	0.796046	0.0106006
Q86TN4	TRPT1	0.795807	0.0104912
A0A0J9YW36	STMN3	0.794847	0.0100616
P14635	CCNB1	0.794326	0.00983477
A0A024RCM2	ZNF184	0.794296	0.00982157
Q15036	SNX17	0.794231	0.00979368
Q9H0U9	TSPYL1	0.794227	0.00979214
A0A024RCW8	DOM3Z	0.794221	0.00978935
O95382	MAP3K6	0.793537	0.00949946
Q8NFZ5	TNIP2	0.793478	0.009475
A0A1B0GTQ2	RAB34	0.79249	0.00907015
Q8TDM6	DLG5	0.792275	0.00898389
A0A126LAV9	U14	0.792079	0.00890625
P62306	SNRPF	0.791084	0.00851984
Q0P6H9	TMEM62	0.790766	0.00839958
E5RFX8	CCNC	0.790756	0.00839585
A0A024R9Y6	GNL3L	0.790744	0.00839142
P51530	DNA2	0.790674	0.00836479
A6ZKI3	FAM127A	0.790441	0.00827808
Q9UBU8	MORF4L1	0.789802	0.00804371
E9PK91	BCLAF1	0.789517	0.00794107
B3KQF4		0.788826	0.00769656
A0A024QZ47	APPBP2	0.788655	0.00763725
A0A024R3H2	SORL1	0.787989	0.00740957
A4PIV7	SYT-SSX1	0.787705	0.00731424
Q5BJF6	ODF2	0.787698	0.00731205
P41134	ID1	0.78756	0.00726596
B4DZC9		0.787081	0.00710876
B4DLY2		0.786453	0.00690694
Q8TEY7	USP33	0.786042	0.00677749
Q15834	CCDC85B	0.785714	0.00667578
A0A024QZ09	OTUD5	0.785575	0.006633
Q7Z7G8	VPS13B	0.784857	0.00641611
A0A024R466	ITM2C	0.78439	0.00627854
B3KMS0		0.784314	0.00625623
Q9NW38	FANCL	0.784141	0.00620609
Q7Z5H3	ARHGAP22	0.784034	0.00617512
Q8N3Z6	ZCCHC7	0.783691	0.00607719
Q9HBU6	ETNK1	0.783317	0.00597192
Q86XQ3	CATSPER3	0.783103	0.00591231
A0A024R9A9	UBE2T	0.78297	0.00587548
A8K9Y9		0.782912	0.00585953
A1XBS5	FAM92A1	0.782609	0.00577665
Q9NVF7	FBXO28	0.78253	0.00575521
Q8NA72	POC5	0.782218	0.00567135
A8K5A6		0.781996	0.00561226
A8K4B4		0.78128	0.00542551
Q9UBG0	MRC2	0.781136	0.00538852
D3DRP5	C9orf19	0.780968	0.00534586
H0YH20	BCKDHA	0.780859	0.00531818
A0A024RDZ1	RP11-484I6.3	0.780072	0.00512264
Q8IXW5	RPAP2	0.779947	0.00509215
Q9H4I9	SMDT1	0.779177	0.00490801
A0A024RDQ5	GTF3A	0.77907	0.00488278
O00762	UBE2C	0.778751	0.00480866
Q96EI5	TCEAL4	0.778605	0.00477483
Q9NXJ5	PGPEP1	0.778257	0.00469556
Q9HC98	NEK6	0.777778	0.0045881
B2RBG2		0.777681	0.00456665
Q96LW4	PRIMPOL	0.777388	0.00450238

(continued on next page)

supplementary table3 (continued)

Uniprot Accession	Gene Symol	Fold Change	P value
Q8WXI2	CNKSR2	0.776971	0.00441212
Q8WUY9	DEPDC1B	0.776699	0.00435415
Q8NBT0	POC1A	0.776449	0.00430144
A0A024R110	DAZAP2	0.775879	0.00418348
E7EVH7		0.775424	0.00409117
P22413	ENPP1	0.775233	0.00405312
Q8IW19	APLF	0.774926	0.00399238
P51610	HCFC1	0.774561	0.00392138
Q16666	IFI16	0.773143	0.00365567
A0A024R6E4	C14orf118	0.772201	0.00348795
P04731	MT1A	0.771626	0.00338911
Q9H6K5	PRR36	0.771429	0.00335567
P32241	VIPR1	0.771231	0.00332246
B2R636		0.769442	0.00303552
A2RU78	KIF3C	0.769369	0.00302436
B7Z7L8		0.768194	0.00284856
P12109	COL6A1	0.767007	0.00268024
Q9NSB8	HOMER2	0.765796	0.00251775
Q6NTE8	C5orf45	0.764205	0.00231751
A0A024R6A0	ARG2	0.764123	0.00230763
Q06481	APLP2	0.763726	0.00226008
Q8IV53	DENND1C	0.763492	0.00223255
A0A024R1E6	PRAME	0.763184	0.0021967
A0A024R3G4	SC5DL	0.763063	0.00218276
F8WEC0	MMADHC	0.762679	0.00213906
A8K9A5		0.762611	0.00213138
A7BI36	RRBP1	0.762032	0.0020671
B3KSP9		0.761553	0.00201521
P14854	COX6B1	0.761324	0.00199087
A0A024RAP2	HMGCR	0.76115	0.00197249
B7Z1P2	FBXO44	0.760923	0.00194885
Q9H467	CUEDC2	0.760261	0.00188115
Q460N5	PARP14	0.760138	0.0018688
Q9Y6B2	EID1	0.759746	0.00182996
A7E2A6	TENC1	0.759473	0.0018033
A8K3H2		0.759296	0.00178619
B2R6E2		0.757119	0.0015877
A0A024R5Z0	TCF12	0.756951	0.00157321
Q3KRA9	ALKBH6	0.75653	0.00153749
B3KY59		0.756288	0.00151729
Q9H8M1	COQ10B	0.756098	0.00150156
Q9HCD6	TANC2	0.755013	0.00141471
P01583	IL1A	0.754275	0.00135825
A9QQ22	WASH	0.753833	0.00132536
Q86U70	LDB1	0.753052	0.00126902
A0A090N8N0	LOC340312	0.751687	0.00117576
E9PSH4	MAF1	0.751618	0.00117123
Q9BXL8	CDCA4	0.751332	0.00115255
A0A0S2Z3S5	GNAS	0.751318	0.00115164
P27469	G0S2	0.747687	0.00093691
P51636	CAV2	0.746933	0.000897104
O95067	CCNB2	0.745698	0.000835268
B2R6V2		0.743483	0.000733904
Q15773	MLF2	0.741379	0.000648104
P23458	JAK1	0.740775	0.000625194
O15031	PLXNB2	0.738899	0.000558679
B2RBI4		0.738703	0.000552133
B7Z8Z2		0.737619	0.000517084
P49796	RGS3	0.737073	0.000500184
A8K9U1		0.736554	0.000484623
Q9P2N7	KLHL13	0.736395	0.00047992
A0A024RC10	hCG_1740677	0.735523	0.000454972

(continued on next page)

supplementary table3 (continued)

Uniprot Accession	Gene Symol	Fold Change	P value
Q9Y5W7	SNX14	0.732997	0.000389213
Q8TB96	ITFG1	0.732663	0.000381195
Q96ST8	CEP89	0.731853	0.000362375
Q9BR77	CCDC77	0.73166	0.000358021
O95273	CCNDBP1	0.72931	0.000308685
P58335	ANTXR2	0.726968	0.000265775
P48307	TFPI2	0.726399	0.000256202
A0A024R7M1	RFXANK	0.725604	0.000243372
A0A087WVP1	FAT1	0.725581	0.000243018
E9PR30	FAU	0.725379	0.000239847
B6CAV5	KIR2DS5	0.725256	0.000237943
Q2NL68	PROSER3	0.725151	0.000236331
B2R7K0		0.725049	0.000234765
O75496	GMNN	0.724878	0.000232172
A0A024R416	USP37	0.724167	0.000221666
Q9UJJ7	RPUSD1	0.722411	0.000197542
Q9NZN4	EHD2	0.722222	0.0001951
P60520	GABARAPL2	0.72164	0.000187733
A0AUJ2	PIG58	0.720383	0.000172707
Q9Y6H1	CHCHD2	0.719807	0.000166189
Q9Y2U9	KLHDC2	0.71978	0.000165894
Q9BZK3	NACAP1	0.719711	0.000165125
D3DR37	CEP55	0.717431	0.000141649
A8K2V5		0.716858	0.000136247
A0A024R9B0	CCNE2	0.716769	0.000135429
Q0D2I6	FEZ2	0.713656	0.000109429
O60673	REV3L	0.70691	6.81E-05
Q9NX12		0.706379	6.56E-05
O75386	TULP3	0.705989	6.37E-05
P19021	PAM	0.70572	6.25E-05
A0A024R1S2	CDC6	0.705187	6.02E-05
H7C3I1	ST13	0.704961	5.92E-05
O43405	COCH	0.704	5.52E-05
B2R4N3		0.698502	3.68E-05
Q9UI14	RABAC1	0.695616	2.96E-05
Q8NDG6	TDRD9	0.693738	2.57E-05
Q8IY63	AMOTL1	0.691264	2.12E-05
Q7Z4F1	LRP10	0.691021	2.08E-05
B4DL54	CHURC1-FNTB	0.690759	2.04E-05
Q9HB72		0.690323	1.97E-05
A0A096LP99	PRAMEF18	0.687589	1.59E-05
A0A0U1RQM0		0.687438	1.57E-05
Q8N5Y2	MSL3	0.687057	1.52E-05
Q59G84		0.68617	1.42E-05
A0A024R8B0	ZYG11BL	0.685252	1.32E-05
P0C7M4	RHOXF2B	0.67611	6.24E-06
Q9BS16	CENPK	0.674624	5.50E-06
P43360	MAGEA6	0.666667	2.77E-06
O14958	CASQ2	0.661801	1.79E-06
P41970	ELK3	0.657546	1.22E-06
K7ES00	H3F3B	0.652444	7.56E-07
Q9P0P0	RNF181	0.651351	6.81E-07
B3KNH6		0.648542	5.21E-07
Q16466		0.647702	4.80E-07
H7C4W4	FSTL1	0.645051	3.71E-07
B7Z4N1		0.641295	2.56E-07
X6R7X0	TCEANC2	0.641148	2.52E-07
Q14CZ0	C16orf72	0.640786	2.43E-07
B3KQA0		0.633855	1.20E-07
B9EJA8	MRC1L1	0.625811	5.11E-08
Q9BV57	ADI1	0.618522	2.30E-08
B2RBV5		0.612873	1.21E-08

(continued on next page)

supplementary table3 (continued)

Uniprot Accession	Gene Symol	Fold Change	P value
O15304	SIVA1	0.610169	8.84E-09
P84101	SERF2	0.608929	7.65E-09
Q6IAP2	PNMA1	0.604874	4.73E-09
Q9P2B7	CFAP97	0.597701	1.98E-09
B2R6N3		0.597665	1.97E-09
W0S1J7	Pe7Fe14	0.589892	7.38E-10
O00767	SCD	0.574675	9.68E-11
A0A024R219	ZA20D2	0.569873	4.94E-11
Q8N6I1	EID2	0.569444	4.65E-11
P43356	MAGEA2	0.562372	1.67E-11
Q86WV6	TMEM173	0.550643	2.84E-12
A0A024R838	hCG_20884	0.549632	2.43E-12
Q96LD1	SGCZ	0.547801	1.82E-12
Q9HD64	XAGE1A	0.530159	1.00E-13
J3KSZ0	EIF4A1	0.4675	3.47E-19
A6NFQ7	DPRX	0.328079	7.81E-40

Tables S4. The 26 overlapping proteins between USP35-upregulated proteins and shUSP35-downregulated proteins.

supplementary table4

Differential expressed proteins by iTARQ coupled LC-MS/MS analysis

Uniprot Accession	Gene Symol	USP35 VS. EV		shUSP35 VS. shNC	
		Fold Change	P value	Fold Change	P value
Q8NFZ5	TNIP2	2.5133	3.0243E-12	0.793478	0.009475
Q96GD4	AURKB	2.33068	1.5852E-10	0.797798	0.0114331
Q7Z406	MYH14	1.98886	2.2395E-07	0.809361	0.0184613
P84022	SMAD3	1.78599	1.3517E-05	0.810362	0.0192126
Q9NWW7	C2orf42	1.78356	1.4177E-05	0.827586	0.0367438
A0A024R6A0	ARG2	1.74413	3.0551E-05	0.764123	0.00230763
O00506	STK25	1.69651	7.6021E-05	0.803648	0.0146319
Q14681	KCTD2	1.63932	0.00022153	0.800182	0.0126561
A1A5C5	RRBP1	1.62918	0.00026694	0.799122	0.012099
Q08999	RBL2	1.58171	0.00063033	0.803109	0.0143082
H7C3I1	ST13	1.55764	0.00096552	0.704961	5.9191E-05
Q9UBG0	MRC2	1.54963	0.0011111	0.781136	0.00538852
B0QYU2	CXorf39	1.51111	0.00215964	0.800238	0.0126857
A6NFQ7	DPRX	1.49154	0.00300536	0.328079	7.807E-40
P04066	FUCA1	1.49045	0.00306073	0.808134	0.0175747
Q06481	APLP2	1.4786	0.00372882	0.763726	0.00226008
Q9Y654	CBX1	1.45324	0.00565224	0.818489	0.0263212
A0A0J9YW36	STMN3	1.43563	0.00750375	0.794847	0.0100616
A0A0S2Z455	SERPINI1	1.41735	0.0100196	0.827521	0.0366588
Q15506	SPA17	1.40347	0.0124358	0.807903	0.0174119
Q9NXJ5	PGPEP1	1.39506	0.0141533	0.778257	0.00469556
Q9NWM3	CUEDC1	1.39381	0.0144267	0.817013	0.0248879
P55085	F2RL1	1.3891	0.0155013	0.817869	0.0257113
A0A024R7M1	RFXANK	1.34286	0.0307483	0.725604	0.00024337
A0A024R3H2	SORL1	1.34177	0.0312343	0.787989	0.00740957
D3DS14	FLJ10357	1.33062	0.0366218	0.822993	0.0311232

Tables S5. Correlation between expression levels of USP35 and the clinical features.

Supplementary Table 5

Correlation between expression levels of **USP35** and the clinical features.

Clinicopathogical parameters	n	IHC score (Mean ± Standard deviation)	*p*
Age at diagnosis (years)			0.8213[a]
<65	33	4.40±2.35	
≥65	12	4.23±1.92	
Sex			0.2973[a]
male	24	4.13±1.86	
female	13	4.90±2.95	
Primary tumor size			0.6529[a]
<5cm	33	4.45±2.40	
≥5cm	12	4.10±1.72	
TNM stage			
Ⅰ	20	3.45±1.35	
Ⅱ	11	3.34±2.15	*P*<0.9813[b]
Ⅲ-Ⅳ	14	6.45±1.92	*P*<0.0001[b]

a. Student t test; b. One-way ANOVA.

Tables S6. Correlation between expression levels of RRBP1 and the clinical features.

Supplementary Table 6

Correlation between expression levels of **RRBP1** and the clinical features.

Clinicopathogical parameters	n	IHC score (Mean ± Standard deviation)	*p*
Age at diagnosis (years)			0.9221[a]
<65	33	5.37±1.51	
≥65	12	5.31±2.37	
Sex			0.3117[a]
male	32	5.20±1.63	
female	13	5.80±2.07	
Primary tumor size			0.2933[a]
<5cm	33	5.52±1.59	
≥5cm	12	4.90±2.14	
TNM stage			
Ⅰ	20	4.97±1.16	
Ⅱ	11	4.90±1.96	*P*<0.9919[b]
Ⅲ-Ⅳ	14	6.50±1.87	*P*<0.0188[b]

a. Student t test; b. One-way ANOVA.
